# The Regulatory T Cell Lineage Factor Foxp3 Regulates Gene Expression through Several Distinct Mechanisms Mostly Independent of Direct DNA Binding

**DOI:** 10.1371/journal.pgen.1005251

**Published:** 2015-06-24

**Authors:** Xin Xie, Michael J. T. Stubbington, Jesper K. Nissen, Kristian G. Andersen, Daniel Hebenstreit, Sarah A. Teichmann, Alexander G. Betz

**Affiliations:** 1 MRC Laboratory of Molecular Biology, Cambridge, United Kingdom; 2 European Molecular Biology Laboratory-European Bioinformatics Institute (EMBL-EBI), Hinxton, United Kingdom; University of Georgia, UNITED STATES

## Abstract

The lineage factor Foxp3 is essential for the development and maintenance of regulatory T cells, but little is known about the mechanisms involved. Here, we demonstrate that an N-terminal proline-rich interaction region is crucial for Foxp3’s function. Subdomains within this key region link Foxp3 to several independent mechanisms of transcriptional regulation. Our study suggests that Foxp3, even in the absence of its DNA-binding forkhead domain, acts as a bridge between DNA-binding interaction partners and proteins with effector function permitting it to regulate a large number of genes. We show that, in one such mechanism, Foxp3 recruits class I histone deacetylases to the promoters of target genes, counteracting activation-induced histone acetylation and thereby suppressing their expression.

## Author Summary

The suppressive activity of regulatory T cells provides the immune system with a mechanism to prevent detrimental immune responses, such as autoimmunity, attack of the beneficial commensal microbiota and rejection of the fetus. Intriguingly, expression of a single lineage factor Foxp3 is sufficient to completely reprogram T cells from a pro-inflammatory to a suppressive phenotype. Here, we show that Foxp3 alters the expression of thousands of genes through several independent mechanisms. In many cases, its own ability to bind to DNA appears to be dispensable, but rather it binds indirectly to the DNA by interaction with other transcription factors. Foxp3 then in turn recruits other proteins that affect gene expression through chromatin modification. For example, Foxp3 indirectly binds to the IL-2 promoter via interaction with the transcriptional activators c-Rel, AML-1 and NFAT. This leads to the Foxp3 mediated recruitment of class I histone deacetylases HDAC1, 2 and 3, which in turn counteracts the activation-induced hyper-acetylation of the promoter, thereby switching the gene off. In a way, Foxp3 hijacks pre-existing regulatory mechanism to reverse the transcriptional expression status of the target gene. By dissecting Foxp3 on a molecular level, we also show that this is only one of several independent mechanism utilised by Foxp3.

## Introduction

T_Reg_ cells, a subtype of CD4^+^ T cells, provide the immune system with a checkpoint preventing detrimental immune responses [1]. They not only avert the development of autoimmunity and preclude the rejection of the fetus by the maternal immune system [2–4], but also modulate the immune system to counteract excessive inflammation in response to pathogens and commensal microorganisms [5]. The lineage factor Foxp3, a member of the forkhead winged helix family of DNA binding factors, plays a key role in both T_Reg_ cell lineage commitment and T_Reg_ cell maintenance [6, 7]. Expression of Foxp3 is essential for T_Reg_ cell differentiation irrespective of whether the T cells commit to the T_Reg_ lineage during their development in the thymus or if they are induced in the periphery at a later stage [8]. Foxp3 acts in conjunction with specific changes in DNA-methylation to drive full commitment to the T_Reg_ identity [9] Lack or loss of functional Foxp3 leads to widespread autoimmunity caused by the absence of T_Reg_ cells [2, 10]. In the absence of Foxp3 it is possible for ‘would-be’ T_Reg_ cells to develop but these lack suppressive activity and have a more Th2-like phenotype [11].

Despite its importance in the development and maintenance of T_Reg_ cells, the molecular mechanisms involved in Foxp3-mediated changes of the transcriptional program governing T_Reg_ cell lineage commitment are poorly understood. Foxp3 has been shown to form macromolecular complexes, yet their exact composition is unknown [12]. A study using an unbiased proteomics approach suggests that Foxp3 interacts with in excess of 300 proteins [13]. It is unlikely that all of them bind Foxp3 at the same time and the composition, dynamics and function of complex formation remain to be resolved. For now, studies addressing individual interaction partners point to the complexity of Foxp3-mediated regulation of gene expression.

The forkhead domain (FKH) of Foxp3 has been shown to be important in its homo-dimerization and DNA binding [14]. The consensus sequence for Foxp3 DNA-binding appears to be rather degenerate and, as such, it has been proposed that Foxp3’s FKH has only low intrinsic affinity for DNA and therefore relies on hetero-dimerization with other transcription factors [15–17]. Indeed, Foxp3 interacts with nuclear factor of activated T cells (NFAT) through its FKH and forms heterodimers with Foxp1 through interactions with its leucine dimerization domain [16, 18]. It also interacts with Ikaros family member Eos and AML1 [19, 20], as well as the transcription factors GATA3, c-Rel and RORγT [13, 21, 22]. Further complexity is introduced by Foxp3's reported association with chromatin modifiers such as the class II histone deacetylases (HDAC) 7 and 9 as well as the histone acetyltransferase TIP-60 [23]. In most cases, the modes of interaction and their functional roles remain to be resolved.

Ectopic expression of Foxp3 in T_H_ cells (T_H_::Foxp3) is sufficient to induce a T_Reg_ cell-like phenotype [24]. These T_H_::Foxp3 cells alter their transcriptional program to up-regulate many genes characteristic of T_Reg_ cells such as *Cd25*, *Ctla4*, *Tnfrsf18* and *Lag3* and to suppress pro-inflammatory genes such as *Il2* [6, 24, 25]. Notably, T_H_::Foxp3 cells not only have suppressive activity *ex vivo*, but can also compensate for the absence of T_Reg_ cells *in vivo* [26]. Thus, ectopic expression of Foxp3 in T_H_ cells provides us with a system to study the transcriptional changes mediated by this protein in the lineage commitment of T_Reg_ cells.

Here we explored how Foxp3 regulates gene expression on a molecular level. We analysed the transcriptomes of T_H_ cells ectopically expressing either wild type or mutant Foxp3 and showed that Foxp3 is involved in the activation and suppression of genes equally. We find that Foxp3 lacking the DNA binding FKH still retains the capability to regulate a large number of its target genes while, in contrast, deletion of a proline-rich interaction domain (ProR) leads to a loss of most Foxp3-mediated gene regulation. Due to its extremely high proline content, this region is disordered, making it impossible to deduce any hints as to potential functional domains based on structural predictions. Instead, we used a variety of comparative genomics approaches to determine subdomains or motifs that are evolutionarily conserved due to their functional importance. Our analysis of the effects of deletion of these motifs on Foxp3-mediated transcriptional regulation suggests that subdomains within the ProR of Foxp3 are involved in several independent mechanisms of transcriptional control, regulating distinct sets of genes; one such mechanism is the recruitment of class I HDACs to Foxp3 target genes such as *Il2* and *Ifng*. In summary, our data suggest that Foxp3 acts as a scaffold for the recruitment of interaction partners with diverse functions. Some of them target the complex to the relevant control elements of the genes to be regulated, while others provide the complex with effector functions such as the ability to modify chromatin to modulate gene expression.

## Results

### Identification of T_Reg_ relevant, Foxp3-regulated genes

To study the role of the functional domains of Foxp3 in gene regulation, we transduced naive CD4^+^CD25^-^ T_H_ cells with retroviral expression vectors encoding wild-type Foxp3, Foxp3 mutants or a GFP control. We ensured equivalent protein expression levels of all Foxp3 constructs used here (data shown in the relevant sections). The expression of many Foxp3-regulated genes is known to be influenced by cellular activation [15], so we rested the cells for 48h post transduction before purifying the mRNA for transcriptome analysis by RNA-seq. We obtained, on average, 1.0x10^8^ reads per library with 7.7x10^7^ properly paired reads mapping unambiguously to the mouse genome (GRCm38v75; S1 Table).

We determined the number of reads that mapped to each gene within the mouse genome and used these counts to identify genes that were significantly differentially expressed between samples. We then filtered the lists for biologically relevant changes. The expression level of each gene within a cell population falls within a bimodal distribution (S1A Fig) [27]. One mode contains genes without any expression (not expressed, NE) and those which are only expressed at a very low level (lowly expressed, LE, < 1 transcript per cell). The other mode contains highly expressed genes (HE) and it is this population that has been proposed to be functionally relevant [27]. We classified the gene expression of our samples according to this bimodal distribution (S1B Fig) and used this to filter the lists of differentially expressed genes. For each pairwise comparison, genes that were classified as NE or LE in both conditions were ignored, as they are likely to be representative of transcriptional noise rather than biologically relevant changes [27].

Foxp3 transduced (T_H_::Foxp3) cells are sufficient to compensate for the absence of T_Reg_ cells both *in vitro* and *in vivo* [6, 28] and this provides a tractable system in which to study Foxp3’s mechanisms of gene regulation. However, the transcriptional program of Foxp3-transduced T_H_ cells is different from that of *ex vivo* T_Reg_ cells. We found that 8,128 genes were differentially expressed between naive T_H_ and T_Reg_ cells using existing RNA-seq data [29], whereas 5,136 genes were differentially expressed between T_H_::control and T_H_::Foxp3 cells (S2A Fig). To ensure that we focussed in this study on Foxp3-regulated genes involved in T_Reg_ function we took the intersection of these sets, which represents 2,407 Foxp3-regulated, T_Reg_-relevant genes. Expression of Foxp3 led to upregulation of 1,255 and downregulation of 1,152 T_Reg_ cell relevant genes (S2 Table) indicating a slight preference for Foxp3-upregulated genes within this list (*P* = 0.037, binomial test). The transcriptomic analyses presented here are robust to the choice of the T_Reg_-relevant, Foxp3-regulated gene set; we also performed the same analysis of a gene set defined as above using 3,092 differentially expressed genes between T_Reg_ and naive T_H_ cells from previously described microarray analyses [30], leading to 1,092 T_Reg_-relevant Foxp3-regulated genes. The conclusions drawn from these analogous analyses were unchanged. The overlap between our set of 2,407 genes and this set of 1,092 genes was highly significant (p<1x10^16^, Fisher’s exact test, S2B Fig) and, since RNA-seq is typically more sensitive than microarray analysis [31], we proceeded with the larger gene set.

We analysed the list of 2,407 Foxp3-regulated, T_Reg_-relevant genes to determine the gene ontology (GO) terms that are enriched within it (S3 Table). These included multiple immunology-related terms. Twenty-five percent (609) of the genes are classified as immunology-related by the ImmPort project [32]. This is a significant enrichment of immunology-related genes (*P* = 2.11×10^–69^, Fisher’s exact test).

### Separation of Foxp3’s DNA binding function and its role in complex formation

Previously, much emphasis has been put on the importance of the FKH of Foxp3 since mutations that lead to immunodysregulation, polyendocrinopathy, enteropathy, X-linked (IPEX) syndrome were identified within the FKH [14] and mutations disrupting FKH function caused dysregulation of key T_Reg_ functional genes [18]. Indeed, the FKH has been shown to be involved in key functions of Foxp3 such as DNA-binding, asymmetrical homo-dimerization and hetero-dimerization with NFAT [14, 16, 18].

**Fig 1:**
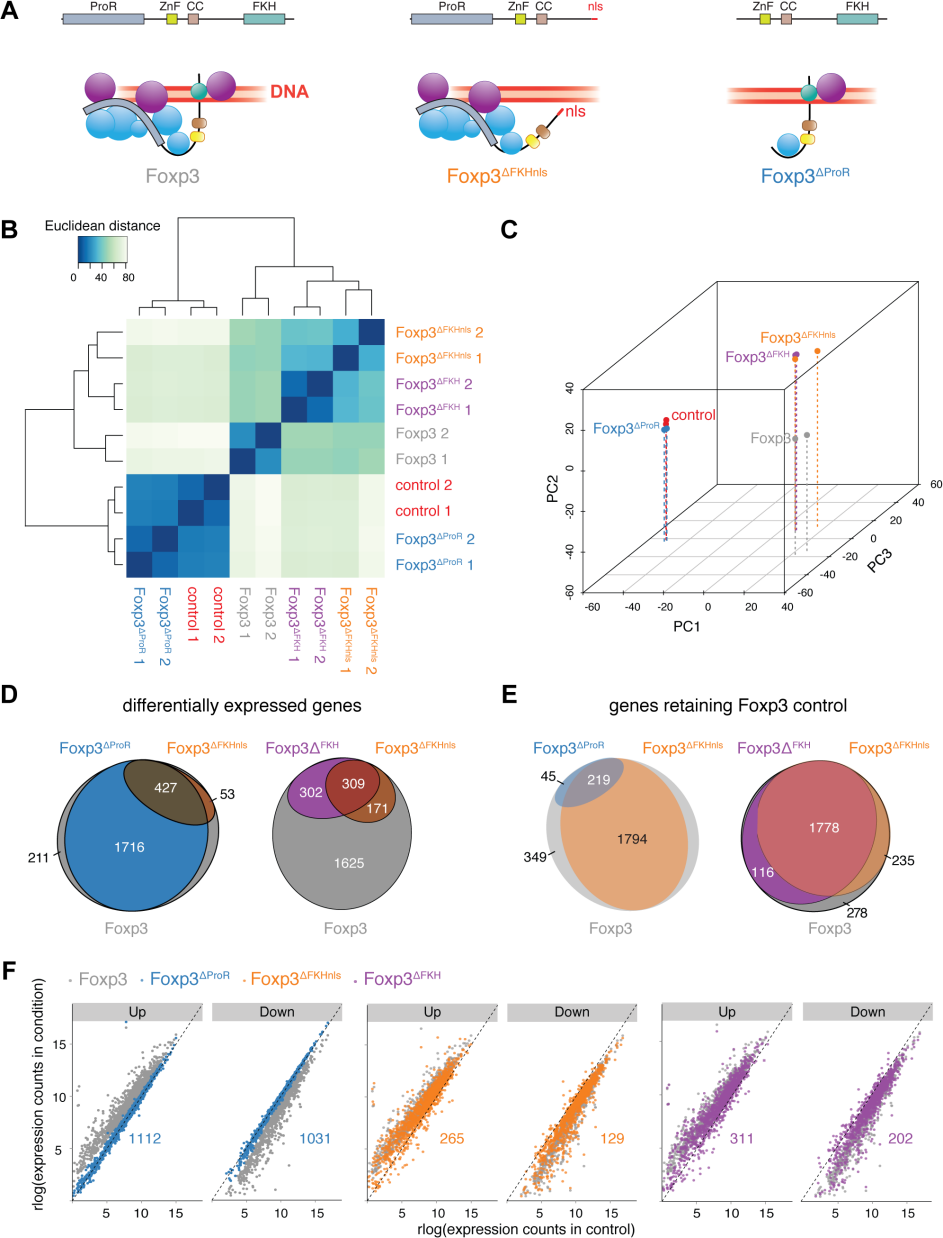
Separation of FKH-mediated DNA binding from the scaffolding function of Foxp3. (A) Model of complex formation and DNA binding of wild type, FKH deletion mutant with an SV40 NLS sequence (Foxp3^ΔFKHnls^) and proline-rich region (exon 1 to exon 3) deletion mutant (Foxp3^ΔProR^) of Foxp3. Spheres represent putative Foxp3 interaction partners with (purple) or without (blue) their own DNA binding capability. (B) Quantification of the similarity in the gene expression profiles of naïve T_H_ cells transduced with expression vectors for wild type (Foxp3), GFP (control) or mutant Foxp3 genes: Foxp3^ΔFKHnls^, Foxp3^ΔFKH^, Foxp3^ΔProR^. Transcriptomes from two independent experiments are shown. Euclidean distances were calculated from regularized log-transformed read counts for Foxp3-regulated genes. (C) Principal-component analysis of the transcriptomes calculated from gene expression data using regularized log-transformed read counts. Color spheres represent individual experiments. PC1, PC2 and PC3 account for 55.4%, 21.1% and 7.22% of the variance, respectively. PC values were calculated using all samples used in this study, but only the samples relevant to this figure are shown here. (D) Venn diagrams showing sets of Foxp3-regulated genes which are differentially expressed (adjusted *P*< 0.05) in a comparison between T_H_::Foxp3 cells and those transduced with other constructs. The set of all Foxp3-regulated genes is represented by a grey ellipse, while colored ellipses represent sets of differentially expressed genes. (E) Venn diagrams showing sets of Foxp3-regulated genes that retain their Foxp3-like control in cells transduced with the indicated constructs. The set of all Foxp3-regulated genes is represented by a grey ellipse, while colored ellipses represent sets of genes retaining Foxp3-like control. (F) Comparison of Foxp3-regulated gene expression in T_H_::control cells and those transduced with other constructs. Each dot represents an individual Foxp3-regulated gene. Genes are separated into those shown to be upregulated by Foxp3 expression and those that are downregulated. Differential gene expression was calculated from the two independent experiments using Wald tests on moderated log2 fold changes as implemented by the DESeq2 R package with *P* values adjusted for multiple testing using the procedure of Benjamini and Hochberg [58]. Numeric annotations on the graphs indicate the number of Foxp3-upregulated and Foxp3-downregulated genes that are dysregulated by each mutant.

It is possible that point mutations within the FKH [18] are more widely disruptive to the structure of Foxp3, and so to separate FKH-mediated DNA-binding of Foxp3 from its role in ProR-mediated complex formation we generated three mutants. In Foxp3^ΔProR^ we deleted exons 1 to 3, which encode the ProR (Fig 1A). Since the FKH contains nuclear localisation sequences (NLS) [33], we made two separate FKH-deletion mutants, one with the entire domain deleted (Foxp3^ΔFKH^) and one in which we added an unrelated SV40-derived NLS to the C-terminus of the protein (Foxp3^∆FKHnls^). Analyses of the transcriptional profiles of Foxp3-regulated genes by hierarchical clustering (Fig 1B) and principal component analysis (PCA) (Fig 1C) illustrate the differences in the transcriptional changes caused by mutation of Foxp3.

When assessing transcriptional changes caused by Foxp3 mutants we considered that enhanced upregulation of Foxp3-induced genes or downregulation of Foxp3-suppressed genes by Foxp3 mutants is unlikely to reflect a failure in Foxp3-mediated regulation. Thus, all those genes that exhibited such changes compared to their expression in TH::Foxp3 cells were classed as retaining Foxp3 control (S2C Fig). Conversely, genes expressed at levels closer to those seen in T_H_::control cells rather than in T_H_::Foxp3 cells were classed as “dysregulated”.

### Transcriptomic analysis highlights key role for Foxp3 ProR

The ProR plays a pivotal role in Foxp3-mediated gene expression control. The transcriptomes of T_H_::Foxp3^∆ProR^ cells clustered with those of the T_H_::control cells, suggesting a dramatic loss in Foxp3-mediated regulation in the absence of the ProR (Fig 1B and 1C) despite having equivalent Foxp3 protein levels as wild-type (S3A Fig). Differential gene expression analysis showed that the majority of relevant, Foxp3-regulated genes (2,143 out of 2,407 genes) were significantly differentially expressed in T_H_::Foxp3^∆ProR^ cells compared with wild-type T_H_::Foxp3 cells (Fig 1D). In contrast, deletion of the FKH domain (Foxp3^∆FKHnls^) led to differential expression of only 480 genes. Analysis of the direction of differential expression (S2C Fig) revealed that only 264/2,407 genes (11%) retained Foxp3 control in T_H_::Foxp3^∆ProR^ cells (Fig 1E), while 2,013 genes (84%) retained Foxp3 control in T_H_::Foxp3^∆FKHnls^ cells. We compared the numbers of Foxp3-upregulated and Foxp3-downregulated genes with the distribution seen within the whole set of Foxp3-regulated, relevant genes (Fig 1F and Table 1). Both Foxp3^∆FKHnls^ and Foxp3^∆FKH^ preferentially dysregulated Foxp3-upregulated genes (P = 1.5×10^–9^ and 1.2×10–4 respectively, binomial tests). Collectively, these data indicate that the ProR plays a crucial role in Foxp3-dependent transcription, whereas the FKH is required for the regulation of fewer genes and appears dispensable in many cases.

**Table 1:** Dysregulation of Foxp3-upregulated and Foxp3-downregulated genes by Foxp3 ProR subdomain mutations.

Condition	Number of dysregulated genes…		*P* value for comparison with all Foxp3- regulated genes
	…upregulated by Foxp3	…downregulated by Foxp3	
T_H_::Foxp3^∆ProR^	1112	1031	0.82
T_H_::Foxp3^∆FKH^	311	202	1.2×10^–4^
T_H_::Foxp3^∆FKHnls^	265	129	1.5×10^–9^
T_H_::Foxp3^∆E1^	12	9	0.67
T_H_::Foxp3^∆m4.2^	95	127	5.7×10^–3^
T_H_::Foxp3^∆m5^	109	115	0.32

*P* values were calculated with binomial tests to compare the distribution of Foxp3-upregulated and—downregulated genes with the distribution within all 2,407 Foxp3-regulated, T_Reg_-relevant genes.

### The role of the FKH and Foxp3-mediated nuclear localisation

**Fig 2:**
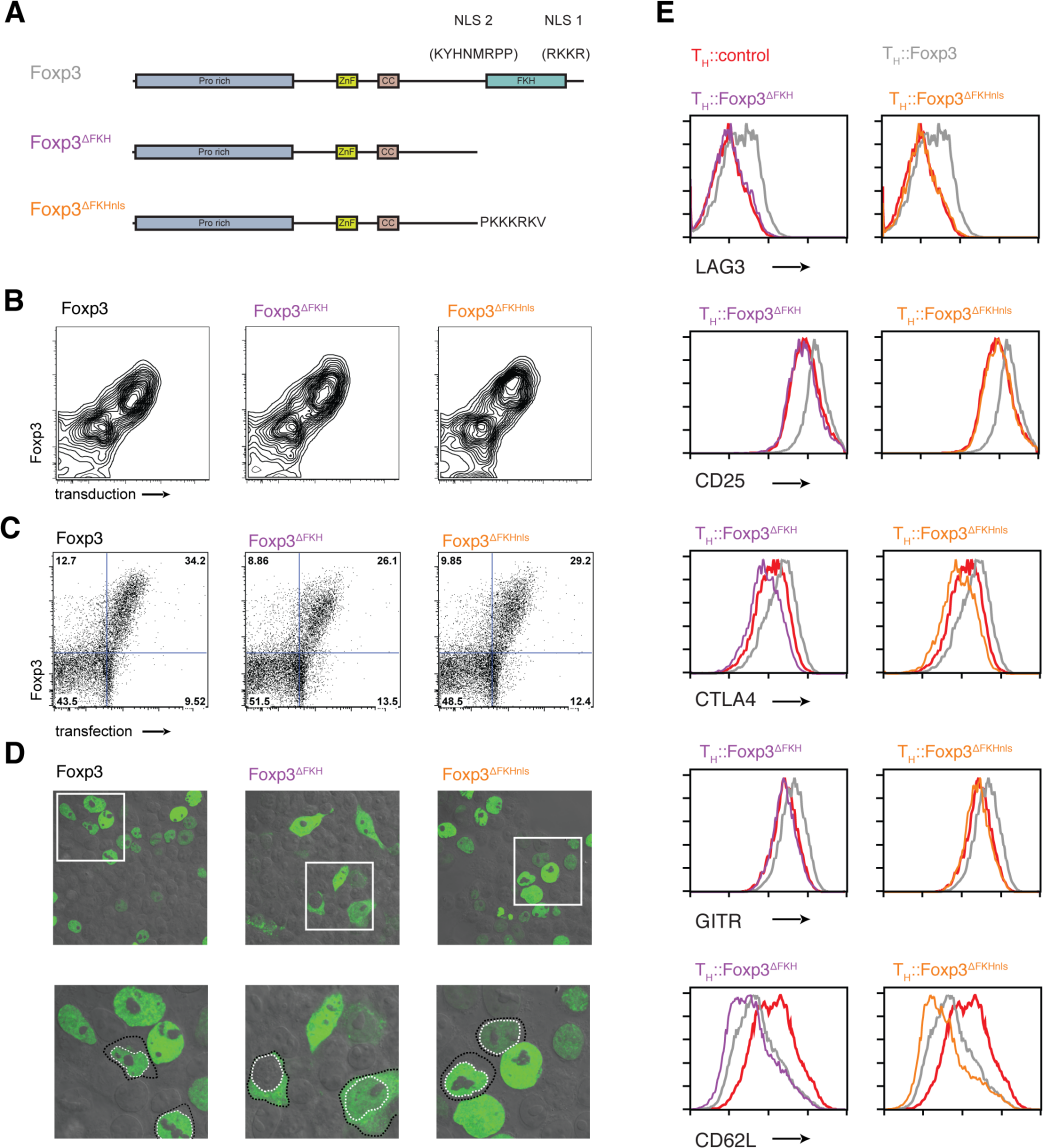
The role of nuclear localisation signals in the forkhead domain of Foxp3. (A) Schematic illustrations of nuclear localization signals and addition of SV40 NLS. (B) Expression of Foxp3, Foxp3^ΔFKH^ and Foxp3^ΔFKHnls^ and the rCD8 reporter in transduced primary T cells. (C) Expression of Foxp3, Foxp3^ΔFKH^ and Foxp3^ΔFKHnls^ and the rCD8 reporter in transfected HEK293 cells. (D) Subcellular localisation of the Foxp3, Foxp3^ΔFKH^ and Foxp3^ΔFKHnls^ stained with alexa488 anti-Foxp3 antibody in transfected HEK293 cells analysed by confocal microscope. Lower panel is a higher magnification of the boxed region in the upper panels with some cells having their nuclei outline with a dotted white line and the cell boundary with a dotted black line. (E) The effect of presence or absence of nls on expression of key molecules on the expression of key Foxp3 target genes. The expression of LAG3, CD25, CTLA4, GITR and CD62L was analysed by flow cytometry 48 h post-transduction in rested T_H_::Foxp3, T_H_::Foxp3^ΔFKH^ and T_H_::Foxp3^ΔFKHnls^ cells. Plots were gated on transduced cells that were rCD8a^+^ and are representative of three independent experiments.

Previous work that disrupted the FKH domain of Foxp3 along with the C-terminal NLS found that this altered the nuclear localisation of the protein and prevented T_Reg_ suppressor function [34]. We used the Foxp3^∆FKHnls^ and T_H_::Foxp3^∆FKH^ mutants to explore the effects of presence or absence of an NLS alongside deletion of the FKH (Fig 2A).

Surprisingly, the transcriptomes of T_H_::Foxp3^∆FKHnls^ and T_H_::Foxp3^∆FKH^ cells clustered with those of the T_H_::Foxp3 cells (Fig 1B and 1C). We found that the majority of genes (1,778 out of 2,407) retained Foxp3-control in both T_H_::Foxp3^∆FKH^ and T_H_::Foxp3^∆FKHnls^ cells (Fig 1D and 1E). Flow cytometry analyses showed that the range of Foxp3 expression in cell populations transduced with each construct was comparable in both primary T cells (Fig 2B) and transfected HEK293T cells (Fig 2C). Furthermore, expression of Foxp3 in T_H_::Foxp3 cells was only slightly higher than that found in primary regulatory T cells (S4 Fig). Intracellular staining of the Foxp3 mutants ectopically expressed in HEK293T cells revealed that the absence of the FKH and NLS did not completely abrogate Foxp3 nuclear localisation, with Foxp3 in both cytoplasm and nucleus (Figs 2D and S5). The sets of genes dysregulated by the two FKH mutants do not overlap exactly (Fig 1D and 1E). This may be due to subtle differences in subcellular localisation of these mutants and suggests that the correct nuclear concentration of Foxp3 is important for gene regulation.

Although the majority of genes are not dysregulated upon deletion of the FKH, this does not mean that this domain is unimportant for Foxp3’s functions. Indeed, we find that genes encoding proteins known to be essential for T_Reg_ function (CTLA4, CD25, GITR, LAG3) are affected by the absence of the FKH in both T_H_::Foxp3^∆FKH^ and T_H_::Foxp3^∆FKHnls^ cells (Fig 2E), albeit the downregulation of CD62L is maintained in both T_H_::Foxp3^∆FKH^ and T_H_::Foxp3^∆FKHnls^ cells.

### Comparative genomics suggests subdomains in ProR

**Fig 3:**
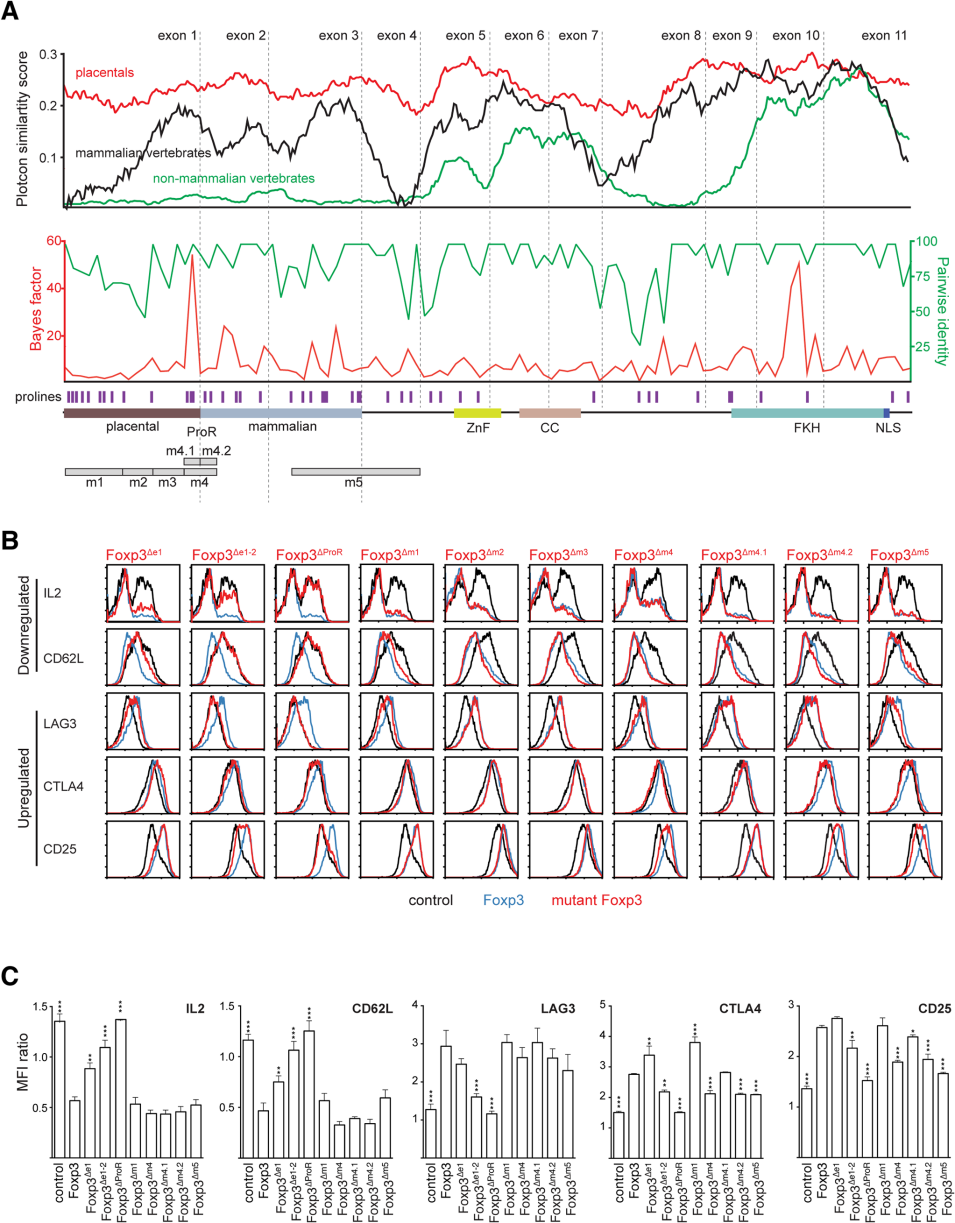
Mutational dissection of ProR of Foxp3 reveals distinct mechanisms of gene regulation. (A) Conservation plot (top) showing the average similarity score at individual amino acid positions from multiple sequence alignments of placental mammalian Foxp3 (red; seven species), non-placental mammalian Foxp3 (black; three species) and non-mammalian vertebrate Foxp3L (green; seven species). The ‘placental’ and ‘mammalian’ halves of the ProR were defined semi-arbitrarily based on the homology across different species. The plots were generated using EMBOSS plotcon with a window size of 20. The REL test as implemented in Datamonkey was used to perform a sliding window analysis (4 codons) of placental Foxp3s with calculated Bayes factors plotted in red (bottom). The average pairwise identity (averaged over 4 amino acids) between Foxp3s is plotted in green (dotted grey lines: exon boundaries). The positions of proline residues are denoted by purple bars. For orientation, a schematic representation of Foxp3 is shown at the bottom. m1-m5 denote regions of interest for which deletion mutants were generated. (B) Flow cytometry analysis of Foxp3 target gene expression in T_H_ cells transduced with wild type Foxp3 (blue), control vector (black) and Foxp3 mutants (red). Foxp3^Δe1^: deletion of exon1; Foxp3^Δe1-2^: deletion of exon 1–2, Foxp3^ΔProR^: deletion of exon 1–3; Foxp3^Δm1^: deletion of region m1; Foxp3^Δm2^, Foxp3^Δm3^, Foxp3^Δm4^, Foxp3^Δm5^: regions m1, m2, m3, m4, m5 were replaced by short stretches of alanines. Foxp3^Δm4.1^ and Foxp3^Δm4.2^ are outlined in S2A Fig. Expression was measured in transduced cells rested 48 h post-transduction (plots were gated on rCD8a^+^ transduced cells and are representative of three independently transduced cell samples in parallel). For IL-2, resting cells were re-stimulated for 12 h with cell stimulation cocktail (PMA and ionomycin with protein transport inhibitor) before staining. (C) Summaries of normalized mean fluorescence intensity (MFI) in (B), shown as ratios of MFI (mean ± SD) of rCD8a^+^ cells/ rCD8a^-^ cells in each sample. Significant differences between T_H_::Foxp3 and cells transduced with the other constructs were determined by one way analysis of variance (ANOVA) followed by Tukey’s post-hoc test(* indicates *P*≤0.05; ** indicates *P*≤0.01; *** indicates *P*≤0.001).

We next sought to identify subregions of the Foxp3 ProR that are likely to be important in its control of gene expression. As the ProR is structurally disordered one cannot discern functional domains based on structural cues and so we developed an alternative strategy using comparative genomics. Evolution tends to conserve functionally important residues [28], clusters of which are indicative of functional subdomains. A comparison of multiple-sequence alignments of the amino-acid sequence of Foxp3 revealed that the sequence homology of orthologs across all vertebrates was restricted to the C-terminal half of the protein which contains the FKH, coiled coil domain (CC) and zinc finger domain (ZnF). The sequence-space occupied by the ProR in mammals was also present in non-mammalian vertebrates although in these species this region is not conserved (Fig 3A, top) and has low proline content (~5% vs ~20%). Stretches of proline-rich content first appeared in non-placental mammals (Fig 3A, top).

A codon alignment of Foxp3 identified stretches of residues where the number of synonymous (silent) mutations significantly exceeded that of non-synonymous (replacement) mutations (Bayes factor). We calculated the moving average of the Bayes factor for the event that regions within Foxp3 are under purifying selection [28], as well as the moving average of pairwise identity. Several regions within Foxp3 had high values for both, indicating that they are under purifying selection (Fig 3A, middle). Notably, apart from a peak in the FKH, the strongest signals of selection were found in the ProR, suggesting that once this region was acquired, it remained under strong evolutionary constraints.

Based on our analyses, the part of the ProR unique to placental mammals can be divided into 4 distinct regions (m1-m4), each of which is framed by prolines (Figs 3A, bottom, and S3B). Regions m1 and m3 have relatively high pairwise identity, but low average Bayes factor. In region m2, both the average pairwise identity and Bayes factor are low, indicating that this region is not under selective pressure. In contrast, region m4 is highly conserved and has a high average Bayes factor, suggesting that it is under strong purifying selection and thus likely to be of functional importance. An additional region, m5 which partially overlaps the ProR, was identified as highly conserved in all mammals.

### Candidate protein analysis suggests distinct roles for ProR subdomains

**Fig 4:**
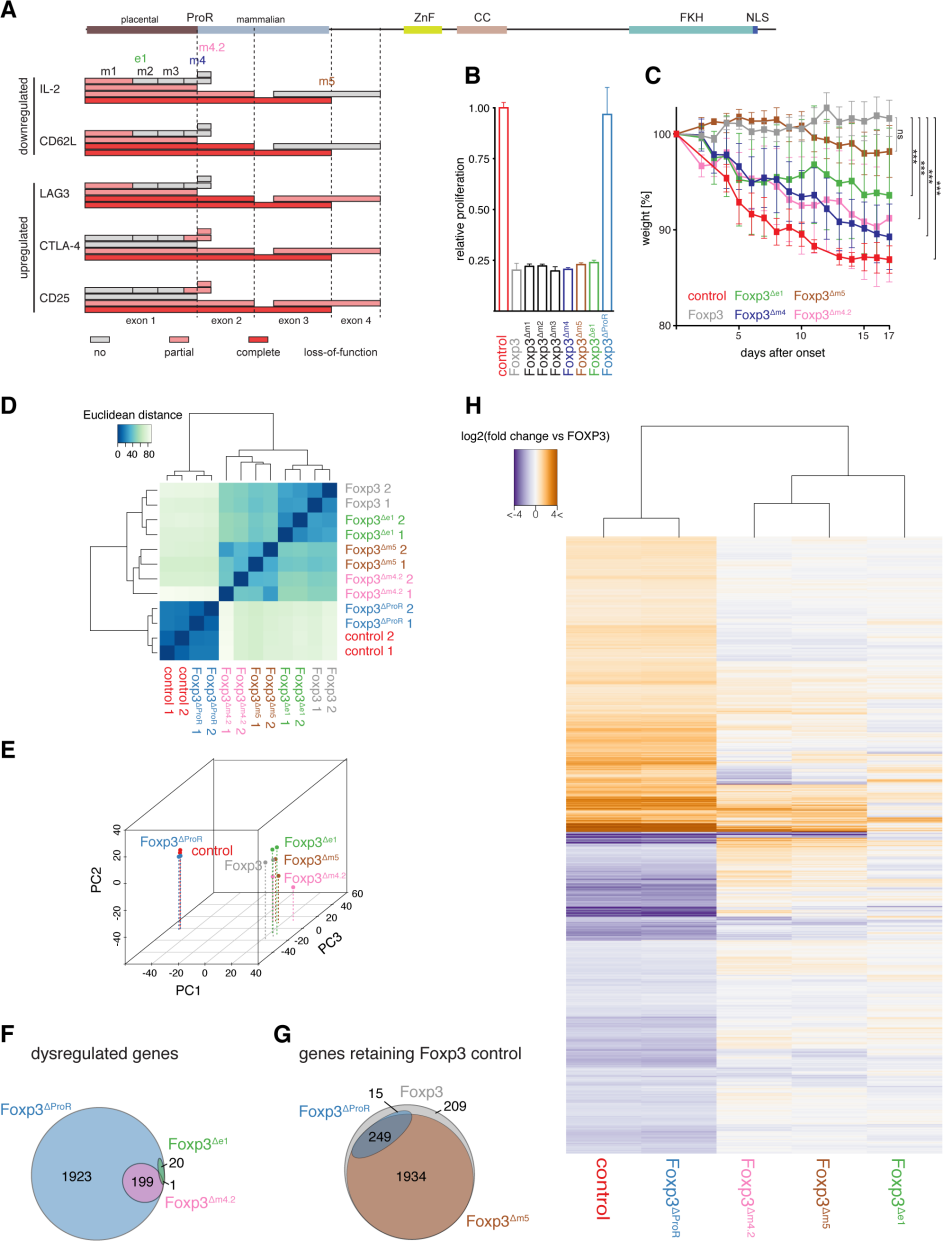
Mutational dissection of ProR of Foxp3 reveals distinct transcriptional programs. (A) A representation of the Foxp3 mutants’ effects on the expression of target genes measured by flow cytometry. Each box maps the region that has been deleted/replaced by stretches of alanines with the fill in color denoting the effect on the examined gene. (grey: no loss of function; light red: partial loss of gene regulation; red: complete loss of gene regulation) A representation of the full length Foxp3 is shown above with the dotted lines marking the exon boundaries. (B) Suppressive activity of Foxp3 or Foxp3 mutant transduced cells were normalized to that of control transduced cells, which were measured as the relative proliferation of co-cultured 'target' CD4^+^CD25^-^ T_H_ cells. Data are shown as means ± SD of three independent experiments. (C) FACS sorted CD4^+^CD25^-^CD45RB^hi^ T_H_ cells were co-transferred with empty vector (control), Foxp3 or Foxp3 mutant transduced CD4^+^CD25^-^ T_H_ cells into weight-matched female C.B.-17 SCID mice. The mean weight of the mice (n≥5 mice per group) was set to 100% the day before the control mice started to lose weight. Each symbol represents the mean weight ± SEM value for each group on each measurement day. *P* values were determined by one-way ANOVA (ns: not significant, *** *P*≤0.001). (D) Quantification of the similarity in gene expression profiles of T_H_ cells transduced with wild type (Foxp3), GFP (control), Foxp3^ΔProR^, Foxp3^Δe1^, Foxp3^Δm4.2^ or Foxp3^Δm5^. Transcriptomes from two independent experiments are shown. Euclidean distances were calculated from regularized log-transformed read counts for Foxp3-regulated genes. (E) Principal-component analysis of the transcriptomes calculated from gene expression data using regularized log-transformed read counts for each Foxp3-regulated gene. Color spheres represent individual experiments. PC1, PC2 and PC3 account for 55.4%, 21.1% and 7.22% of the variance, respectively. PC values were calculated using all samples used in this study, but only the samples relevant to this figure are shown here. (F) Venn diagram showing sets of Foxp3-regulated genes that retain their Foxp3-like control in cells transduced with the indicated constructs. The set of all Foxp3-regulated genes is represented by a grey ellipse while the blue ellipse indicates the set of genes that retain their regulation in T_H_::Foxp3^ΔProR^ and the brown ellipse those genes that retain their regulation in T_H_::Foxp3^Δm5^. (G) Venn diagram showing the set of all Foxp3-regulated genes dysregulated by deletion of the ProR (T_H_::Foxp3^ΔProR^; blue ellipse). The pink ellipse represents those genes that are dysregulated in TH::Foxp3Δm4.2 and the green ellipse those dysregulated in T_H_::Foxp3^Δe1^. (H) Heat-map of log-transformed fold-changes in expression of Foxp3-regulated genes between T_H_::Foxp3 cells and cells transduced with the various Foxp3 mutants. The dendrogram above the plot indicates hierarchical clustering performed using Euclidean distance. Differential gene expression in (F), and (G) was calculated from the two independent transduction experiments shown in (D) and (E) using Wald tests on moderated log2 fold changes as implemented by the DESeq2 R package with *P* values adjusted for multiple testing using the procedure of Benjamini and Hochberg [59].

Based on these findings we generated Foxp3 mutants in which ProR subdomains were either deleted or replaced with short stretches of alanines, ectopically expressed them in T_H_ cells and used flow cytometry to assess their importance in the expression of proteins known to be important in T_Reg_ cell function (Fig 3B and 3C). We also assessed the effect of the removal of exon 1, exons 1 to 2 or the entire ProR. We ensured that the Foxp3 mutants were expressed at similar levels and localized to the nucleus of the transduced cells (S3A Fig). These results are summarized in Fig 4A.

Removal of the ProR led to a complete loss of Foxp3-mediated regulation of all T_Reg_ relevant proteins analysed (Foxp3^ΔProR^). The deletion of the first two exons had a similar effect, although the dysregulation of CTLA4 and CD25 was less marked (Foxp3^Δe1-2^). When we deleted exon 1, which is only conserved in placental mammals, Foxp3-mediated regulation of IL-2, CD62L and LAG-3 was impaired (Foxp3^Δe1^). The regulation of CTLA4 and CD25 remained unaffected by this mutation and ∆m1 (Foxp3^Δe1^, Foxp3^Δm1^). In contrast, replacement of region m4 with a stretch of five alanine residues severely affected Foxp3’s ability to regulate CTLA4 and CD25 (Foxp3^Δm4^), but did not affect the regulation of IL-2, CD62L and LAG3. Replacement of regions m2 and m3 with stretches of alanine residues had no effect on the regulation of any of these proteins. When we replaced region m5, we found that it only affected the expression of candidate proteins upregulated by Foxp3 (Foxp3^Δm5^).

Region m4 spans the junction between exons 1 and 2 but deletion of exon 1 did not dysregulate the proteins affected by removal of region m4. This implies functional importance of the second half of region m4, contained in exon 2 (Fig 4A). Thus we generated mutants with each half of m4 replaced separately by alanines (Foxp3^Δm4.1^ and Foxp3^Δm4.2^). The loss of function observed (Fig 3B and 3C) could be attributed to the four replaced amino acids (LVMV) in the N-terminal region of exon 2. This motif is fully conserved in all placentals (S6A Fig). qPCR analysis of target gene expression (S6B Fig) mirrored protein expression. The lack of overlap between the target genes affected by deletions of exon 1 and subdomain m4.2 suggests that they are regulated by two independent mechanisms (Fig 4A). Combined, these analyses suggest that the ProR mediates T_Reg_ cell-specific gene expression through more than one mechanism.

### ProR subdomains confer distinct T_Reg_ functions

Foxp3^∆ProR^ is unable to confer a T_Reg_ phenotype in both *ex vivo* suppression assays (Fig 4B) and in an *in vivo* model of autoimmune colitis induction (Fig 4C). However, only *in vivo* assessment revealed a functional role for the ProR subdomains (m1 to m5). Alanine replacement of region m4 or subdomain m4.2 led to the most severe impairment of the transduced cells in preventing weight loss (Fig 4C), confirming the importance of the LVMV motif for Foxp3 function. Deletion of region m1 led to a significant impairment of the transduced cells in preventing weight loss, albeit slightly less marked than that observed upon deletion of the entire exon 1. Alanine replacement of region m5 had a milder effect, not reaching statistical significance in our experiments (Fig 4C). While these assays confirm the importance of the subdomains for overall T_Reg_ function, they give no clues about the molecular mechanisms affected. The discrepancy between *ex vivo* and *in vivo* results is likely due to Foxp3 affecting T_Reg_ cell function via multiple mechanisms such as cell-cell interactions, homeostasis, tissue homing and effector function, which might not all be required in *ex vivo* assays.

### Subdomains within the ProR link into distinct transcriptional regulatory mechanisms

A transcriptomic analysis of the effects of the subdomain deletions confirmed that the ProR links into several distinct mechanisms of gene regulation. Hierarchical clustering (Fig 4D) and PCA (Fig 4E) revealed that the transcriptomes of T_H_::Foxp3^Δe1^, T_H_::Foxp3^Δm4.2^ and T_H_::Foxp3^Δm5^ cells were much closer to that of T_H_::Foxp3 than to T_H_::Foxp3^ΔProR^ cells. We examined the distribution of Foxp3-upregulated and Foxp3-downregulated genes within the lists of dysregulated genes for each mutant and compared these distributions with that within all 2,407 Foxp3-regulated genes (Table 1). Foxp3^Δm4.2^ appears to preferentially dysregulate Foxp3-downregulated genes. Deletion of region m5 affects the most genes (224/2407, Fig 4G; Foxp3^∆m5^), yet has the mildest phenotype in the *in vivo* model of autoimmune colitis induction (Fig 4C). In contrast, deletion of exon 1 which affects the least Foxp3 regulated genes results in a more marked phenotype *in vivo* (Fig 4C; Foxp3^Δe1^). Clearly, the number of genes that become dysregulated does not directly correlate with the severity of the *in vivo* phenotype and would require a much more detailed analysis to elucidate the role of the Foxp3 subdomains in the function of T_Reg_ cells. However, our approach allows us to draw firm conclusions regarding the control of sets of genes regulated by Foxp3. Alanine replacement of m4.2 dysregulated 210 genes. The effect of deletion of exon 1 was even smaller with only 21 genes becoming dysregulated in T_H_::Foxp3^Δe1^ cells (Fig 4F; Foxp3^Δe1^). Only one gene dysregulated by Foxp3^∆m4.2^ was also affected by deletion of exon 1 (Fig 4F) and all affected genes were strict subsets of the set of genes dysregulated in T_H_::Foxp3^ΔProR^. This strongly suggests that Foxp3 ProR region utilizes two independent mechanisms to regulate these gene sets.

### Gene ontology enrichment analysis of lists of dysregulated genes

We analysed the lists of genes dysregulated by each Foxp3 mutant to determine the number of immunology-related genes as well as the GO-terms enriched within the lists (Tables 2 and S3). In each case there was a significant enrichment of immunology-related genes. We classified enriched GO terms as immunology-related or non-immunology-related and found that 12.7% of enriched terms were immunology related for the set of all Foxp3-regulated genes. The GO terms enriched within the lists of genes dysregulated by the Foxp3^ΔFKH^ and Foxp3^ΔFKHnls^ mutants contained a significantly higher proportion of immunology-related terms.

**Table 2:** Analysis of immunology-related genes and enriched GO terms within gene lists.

Gene list	Analysis of immune-related genes			Analysis of GO term enrichment		
	Immunology-related genes	Non-immunology genes	Enrichment *P* value^a^	Immunology-related GO terms	Non-immunology GO-terms	*P* value for comparison with all Foxp3-related genes^b^
All Foxp3-regulated, T_Reg_ relevant genes	609 (25.3%)	1798 (74.7%)	2.1×10^–69^	34 (12.7%)	234 (87.3%)	N/A
T_H_::Foxp3^∆ProR^	556 (25.9%)	1587 (74.06%)	7.4×10^–68^	34 (13.2%)	223 (86.8%)	0.78
T_H_::Foxp3^∆FKH^	178 (34.7%)	335 (65.3%)	3.4×10^–41^	26 (26.3%)	73 (73.7%)	2.3×10^–4^
T_H_::Foxp3^∆FKHnls^	154 (39.1%)	335 (65.3%)	3.9×10^–43^	40 (32.3%)	84 (67.7%)	1.2×10^–8^
T_H_::Foxp3^∆E1^	8 (38.1%)	13 (61.9%)	1.8×10^–3^	0	0	N/A
T_H_::Foxp3^∆m4.2^	77 (34.7%)	145 (65.3%)	5.9×10^–19^	5 (7.6%)	61 (92.4%)	0.27
T_H_::Foxp3^∆m5^	95 (42.41%)	129 (57.6%)	1.4×10^–7^	11 (19.0%)	47 (81.0%)	0.16

^a^ *P* values calculated by Fisher’s exact test in comparison with all genes in mouse genome.

^b^ *P* values calculated by Binomial test in comparison with distribution of immunology-related and non-immunology-related terms enriched in set of 2,407 Foxp3-regulated T_Reg_-relevant genes.

### ProR-dependent, FKH-independent regulation of IL2

**Fig 5:**
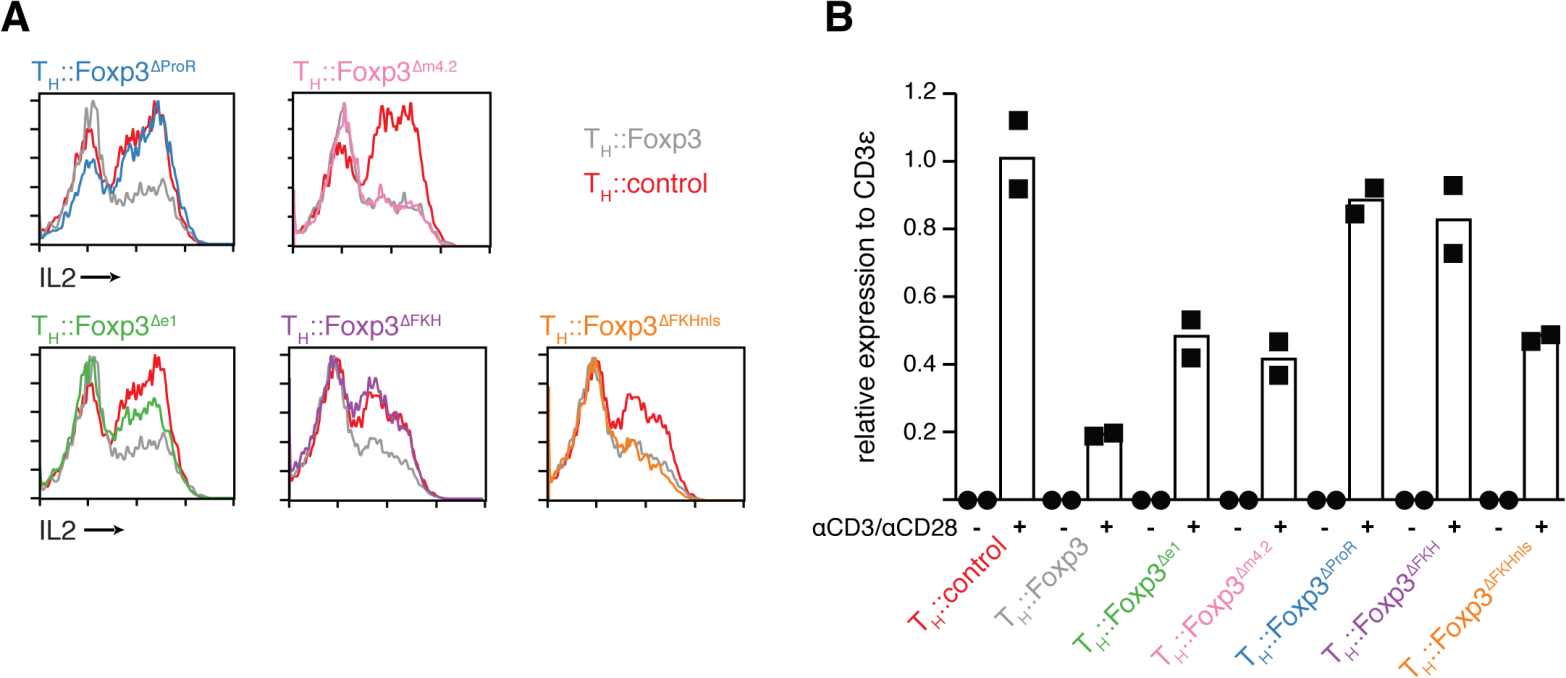
ProR-dependent, FKH-independent regulation of IL-2 upon activation. (A) Activation-induced expression of IL-2 in Foxp3 or Foxp3 mutant transduced cells measured by flow cytometry. Transduced cells were rested and re-activated with cell stimulation cocktail (PMA and ionomycin with protein transport inhibitor) before staining. Data are representative of three independent experiments. (B) Expression of *Il2* mRNA in Foxp3 or Foxp3 mutant transduced cells at resting or re-activation state was determined by quantitative RT-PCR relative to the expression of CD3ε. Circles indicate measurements from resting cells, square measurements from reactivated cells. Height of the bars indicates the mean of the two experiments.

Many genes known to be important for T_Reg_ cell function are also upregulated by activation. For example, *Ctla4* and *Cd25* are upregulated in all activated T_H_ cells [35, 36] but T_Reg_ cells constitutively express these genes irrespective of activation status [1, 6]. To avoid the confounding effects of activation we performed our analyses on resting cells. The expression of activation-induced IL-2 is known to be suppressed by Foxp3 in T_Reg_ cells [11, 37]. While in our transcriptomic data of resting cells *Il2* transcript levels were low in all conditions, activation-induced IL2 expression was suppressed by Foxp3 at the protein level in re-activated cells (Figs 3B and 3C and 4A). We found that the ProR was indispensable for Foxp3-mediated IL2 suppression upon activation (Fig 5A; T_H_::Foxp3^∆ProR^), but contrary to results published previously [18], this suppressive effect was independent of the FKH (Fig 5A; T_H_::Foxp3^∆FKHnls^). We also observed downregulation of *Il2* transcripts by Foxp3 when transduced cells were re-stimulated (Fig 5B). *Il2* downregulation appeared to be very sensitive to changes in nuclear localisation efficiency since it is only dysregulated if the NLS is not present (Fig 5; T_H_::Foxp3^ΔFKH^). Deletion of exon 1 and exon1 to 2 had a partial effect (Fig 5; T_H_::Foxp3^Δe1^), while deletion of another part of the ProR, region m4.2 which had a marked effect on the behaviour of T_H_::Foxp3^Δm4.2^ cells *in vivo* (Fig 4C), had no effect on IL-2 expression (Fig 5; T_H_::Foxp3^Δm4.2^). These results, in combination with the fact that IL-2 plays a central role in the function and behaviour of T_Reg_ cells [37], led us to further investigate the regulation of IL-2 expression as an example of FKH-independent Foxp3-mediated control of gene expression involving the ProR. Foxp3-mediated histone-deacetylation of the *Il2* and *Ifng* promoters has been proposed to downregulate the expression of these genes [24] and Foxp3 appears to interact with various chromatin modifiers. The class I histone-deacetylases (HDAC1, 2, 3), which are ubiquitously expressed in T cells, all appear to associate with the Foxp3 complex [13, 38]. However, little is known about the mode of interaction between Class I HDACs and Foxp3 and the mechanism by which this affects gene expression. The apparent importance of the ProR in the regulation of IL-2 led us to determine whether and how this domain might be involved in the interaction of Foxp3 with class I HDACs.

### Foxp3 ProR directly interacts with class I HDACs

**Fig 6:**
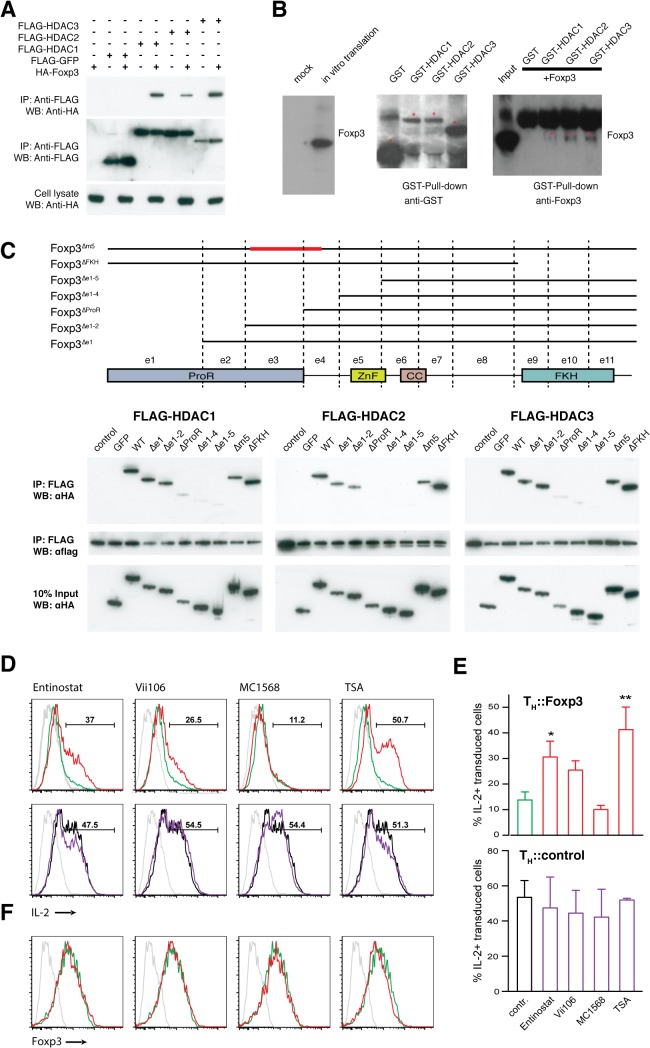
Foxp3 interacts physically with class I HDACs to suppress IL-2. (A) Foxp3 associates with Class I HDACs within cells. HEK293T cells were co-transfected with vectors expressing FLAG-HDAC1, 2, 3 or FLAG-GFP and HA-Foxp3. Cell lysates were immunoprecipitated with anti-FLAG M2 agarose, followed by Western blotting with anti-HA (top), or anti-FLAG (middle) antibodies. The expression of Foxp3 in cell lysate was detected with anti-HA (bottom). (B) Foxp3 directly interacts with Class I HDACs. *In vitro* translated Foxp3 (left) was incubated with bacterially expressed GST-HDAC1, 2, 3 or GST alone. After pull down with GST-affinity beads, the eluent was analyzed by immunoblotting with anti-GST (middle) and anti-Foxp3 (right). Asterisk indicates corresponding GST-fused protein. (C) Interaction of Foxp3 with class I HDACs. (top) Schematic illustration of Foxp3 and the various deletion constructs. ProR: proline-rich domain; ZnF: zinc finger domain; CC: coiled-coil domain; FKH: forkhead domain. Immunoprecipitation of (bottom left) FLAG-HDAC1, (bottom middle) FLAG-HDAC2 and (bottom right) FLAG-HDAC3 co-transfected into 293T cells with HA-tagged Foxp3 or the respective Foxp3 mutants. The lysates were immunoprecipitated with anti-FLAG M2 agarose. Precipitated proteins were probed with anti-HA or anti-FLAG antibodies. (D) Intracellular IL-2 level in (top) T_H_::Foxp3 cells and (bottom) T_H_::control cells in the absence (green, black) or presence (red, purple) of the respective inhibitor (isotype control in grey). Primary T_H_ cells transduced, rested for 40 h and incubated 8 h with Class I HDAC inhibitor Entinostat (1.8 μM), Vii106 (2.4 μM), Class IIa HDAC inhibitor MC1568 (1.5 μM) or pan-HDAC inhibitor TSA (0.05 μM) respectively. The cells were re-activated in the presence of the corresponding HDAC inhibitor for 12 h. Data are representative of three independent experiments. (E) Mean percentage (± SD, n = 3) of cells producing IL-2 in total transduced rCD8a^+^ cells under the various conditions shown in (D). *P* values between control and treatment group were determined by ANOVA followed by Tukey’s post-hoc test (* indicates *P*<0.05; ** indicates *P*<0.01). (F) Intracellular Foxp3 expression level after HDAC inhibitor treatment. T_H_::Foxp3 in the absence (green) or presence (red) of the respective inhibitor (isotype control in grey). For (A), (C), (D) and (F) are representative results of three independent experiments. Blots in (B) are representative of two independent experiments.

We found that the class I HDACs directly interact with the ProR of Foxp3. We confirmed the interaction of these proteins by co-expression of FLAG-tagged HDAC1, 2 or 3 and HA-tagged Foxp3 in HEK293T cells (Fig 6A) and in primary T cells (S7A Fig). Foxp3 could be co-precipitated with all three class I HDACs. To determine whether this interaction was direct we *in vitro* translated Foxp3 protein and incubated it with GST-tagged class I HDACs purified from *E*. *coli*. We found that all three HDACs can directly interact with Foxp3 (Fig 6B).

To map the interaction region, HA-tagged Foxp3 deletion mutants and FLAG-tagged class I HDACs were co-expressed in HEK293T cells (Fig 6C). Deletion of the FKH domain had no effect on the interaction nor did deletion of m5, which has been previously implicated in the interaction between Foxp3 and chromatin modifiers such as HDAC7 and Tip60 [23]. Deletion of ProR (exon 1–3) severely impaired the interaction with class I HDACs while deletion of exons 1–2 had only a partial effect. This might lead one to speculate that exon 3 is crucial for the interaction (Fig 6C). However, we found that deletion of *Foxp3* exon 3 alone had little, if any, effect upon the interaction (S7B Fig). Together, our data suggest an altogether more complex mode of interaction between the ProR of Foxp3 and the class I HDACs. It is possible that the ProR contains multiple HDAC binding sites or that the binding of HDACs is strengthened by other molecules in the complex thus masking the effect of the deletion of their primary interaction site.

### Foxp3’s suppression of IL2 is dependent on activity of class I HDACs

Inhibition of class I HDACs impairs Foxp3-mediated suppression of IL-2 expression. We treated T_H_::Control or T_H_::Foxp3 cells with HDAC inhibitors (Fig 6D and 6E). The class I HDAC inhibitors Entinostat and Vii106 impaired Foxp3-mediated suppression of IL-2. The pan-HDAC inhibitor TSA exhibited an even more marked effect while an inhibitor of class IIa HDACs, MC1568, had no effect on IL-2 regulation (Fig 6D and 6E). These effects were not due to downregulation of Foxp3 as there was no difference in Foxp3 levels between HDAC inhibitor treated and untreated cells (Fig 6F). None of the HDAC inhibitor treatments had an obvious effect on IL-2 in T_H_::control cells (Fig 6D and 6E). Our data shows that inhibition of class I HDACs activity impairs the suppression of IL-2 expression by Foxp3. We therefore decided to investigate whether the interaction between Foxp3 and class I HDACs facilitates their recruitment to the *Il2* promoter. This in turn might explain how Foxp3 binding to the *Il2* promoter leads to its de-acetylation thereby preventing activation-induced upregulation of *Il2* [24]. As we could not exclude off target effects of the HDAC inhibitors, we also knocked down the class I HDACs using a cocktail of HDAC1, 2 and 3 siRNAs. Similar to our findings using the inhibitors we found a marked effect on IL-2 expression (S8 Fig).

### Foxp3 recruitment to the Il2 promoter is FKH-independent

**Fig 7:**
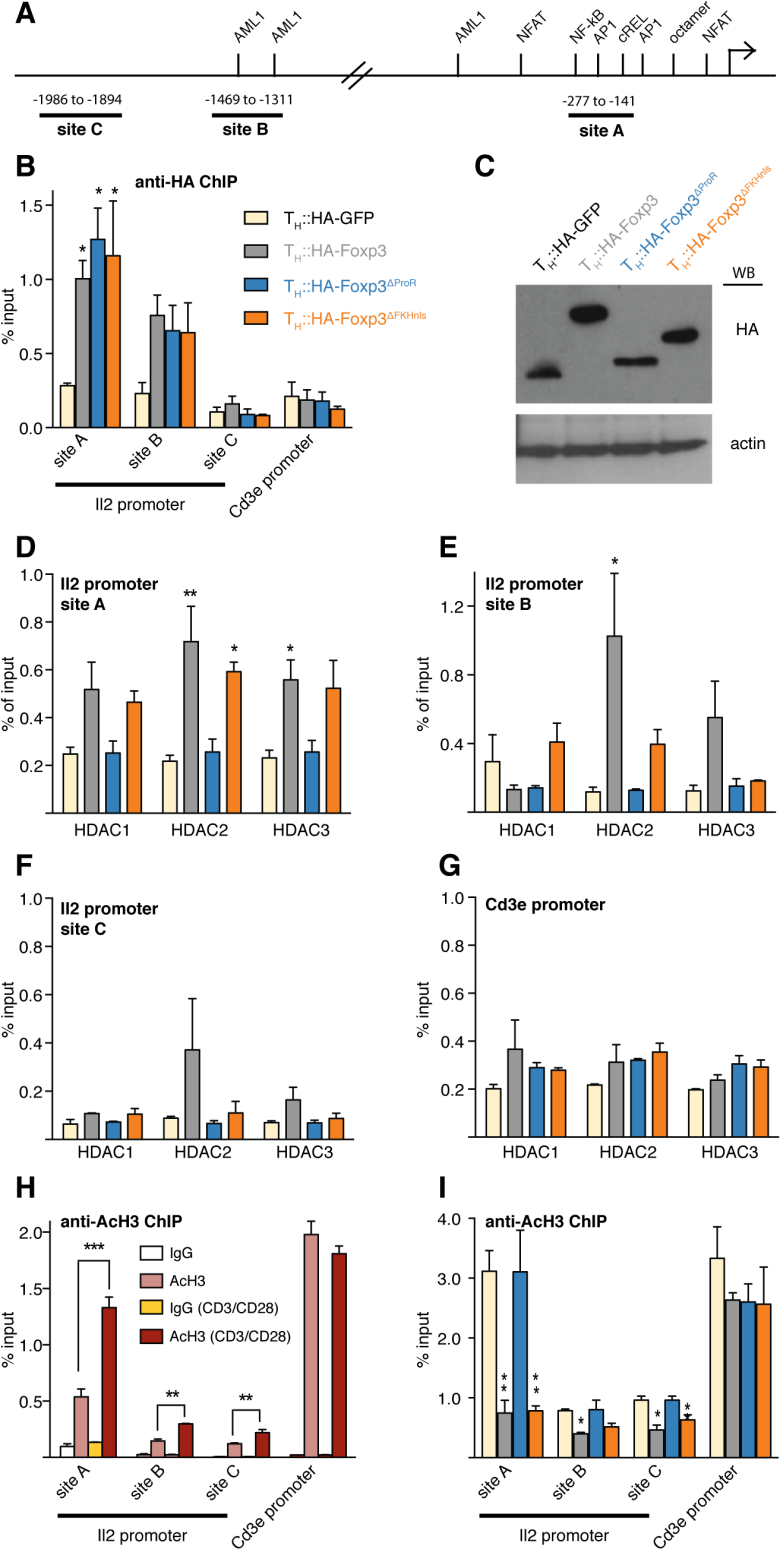
Foxp3 recruits class I HDACs to modulate histone acetylation of the *Il2* promoter. (A) Schematic diagram showing binding sites of well-known transcription factors regulating the *Il2* promoter [19] and the regions analysed in (B to I). (B) Primary T_H_ cells were transduced with retrovirus carrying HA-Foxp3, HA-GFP and HA-tagged Foxp3 deletion mutants. Transduced cells were rested and re-activated by CD3/CD28 for 6 h. ChIP quantitative PCR (qPCR) was used to analyze the binding of HA-GFP, HA-Foxp3 and deletion mutants at the *Il2* promoter using anti-HA antibody. *Cd3ε* promoter was used as negative control. (C) Expression of the respective HA-tagged proteins. Western blot is representative of two independent experiments. (D to G) ChIP-qPCR analysis of the binding of Class I HDACs at different *Il2* promoter sites and *Cd3e* promoter in re-activated transduced T_H_ cells expressing HA-tagged GFP, Foxp3 or the respective deletion mutant—color code as in (B). (H) ChIP-qPCR analysis of *Il2* promoter and *Cd3e* promoter using anti-acetylated histone 3 antibody in primary T_H_ cells, with (dark red) or without (light red) CD3/CD28 activation. Normal rabbit IgG was used as control (white and yellow). (I) ChIP-qPCR analysis of *Il2* and *Cd3e* promoter in re-activated transduced T_H_ cells expressing HA-tagged GFP, Foxp3 or the respective deletion mutant using anti-acetylated histone 3 antibody—color code as in (B). Height of bars represent mean values ± SD. *P* values between T_H_::HA-GFP and T_H_::HA-Foxp3 or the respective Foxp3 mutant transduced cells were determined by one-way ANOVA followed by Tukey’s post-hoc test. P values in (H) between rested and activated T_H_ cells were determined by an unpaired, two-tailed t-test (* indicates *P*<0.05; ** indicates *P*<0.01; ***indicates *P*<0.001). In all cases, data are representative of three independent experiments for each construct.

We found that Foxp3 is recruited effectively to the *Il2* promoter in the absence of the FKH (Fig 7A–7C). This is contrary to the previous suggestion that *Il2* regulation requires cooperative DNA binding via the heterodimerization of the Foxp3 FKH with NFAT [18]. We arbitrarily selected three sites upstream of the *Il2* transcription start site for analysis by ChIP-qPCR (Fig 7A). Site A comprises the proximal part of the *Il2* promoter and contains the binding sites of a multitude of transcription factors while site B is more distal with two AML1 binding sites [19]. Site C is intended to be a control just outside the *Il2* promoter, and contains no obvious transcription factor binding sites. Consistent with previous work [24], we found that the recruitment of Foxp3 to *Il2* promoter sites A and B is activation dependent (S7C–S7E Fig). ChIP-qPCR revealed that upon activation HA-Foxp3^∆ProR^ and HA-Foxp3^∆FKHnls^ were enriched at the *Il2* promoter A and B sites to the same extent as wild type Foxp3, but we found no enrichment at the C site or at the *Cd3*ε promoter which is not regulated by Foxp3 (Fig 7B) [39]. The expression of tagged proteins was confirmed by Western blotting (Fig 7C). While these data do not preclude a role for the FKH in modulating Foxp3 binding to the *Il2* promoter *per se*, our results cast doubt on this being the main mechanism of Foxp3 recruitment. The interaction of Foxp3 with other *Il2*-regulating transcription factors including AP-1, C-Rel and AML1 may be responsible for FKH-independent recruitment [19, 21, 40].

### Foxp3 mediated deacetylation of the Il2 promoter requires ProR

The ProR is necessary for the recruitment of HDACs and subsequent deacetylation of the *Il2* promoter during activation. HDAC1, 2 and 3 were equally enriched at *Il2* promoter site A in T_H_::HA-Foxp3 and T_H_::HA-Foxp3^∆FKHnls^ cells compared with T_H_::HA-GFP cells, but their enrichment was completely impaired in the absence of the ProR (Fig 7D). A similar result was observed at site B for HDAC2 (Fig 7E). As expected, we did not observe a significant enrichment of HDACs at site C nor at the *Cd3*ε promoter (Fig 7F and 7G). Expression levels of HDAC1, 2, 3 were similar in T_H_::HA-Foxp3 and T_H_::HA-GFP control cells (S7F and S7G Fig), so these effects were not due to the upregulation of HDACs by Foxp3.

Consistent with previous findings [41, 42], the *Il2* promoter became hyperacetylated upon activation (Fig 7H). This was especially pronounced at the most proximal site A, but appeared to extend beyond the promoter itself to site C. The constitutively expressing gene *Cd3ε* promoter was highly acetylated regardless of activation state. The presence of HA-Foxp3 or HA-Foxp3^∆FKHnls^ counteracted activation-induced acetylation of the *Il2* promoter, but had no effect on the status of the *Cd3ε* promoter. In contrast, HA-Foxp3^∆ProR^ was unable to abrogate activation-induced acetylation at the *Il2* promoter (Fig 7I).

Importantly, this mechanism of transcriptional control by Foxp3 is not unique to *Il2*; we also observed analogous results at the promoter of the gene encoding IFN-γ. As was the case for *Il2*, Foxp3-mediated suppression of *Ifng* in activated T cells was lost upon deletion of the FKH domain but recovered upon addition of an NLS (S9A Fig). Both HA-Foxp3^∆ProR^ and HA-Foxp3^∆FKHnls^ were equally recruited to the *Ifng* promoter though at slightly lower levels than HA-Foxp3 (S9B Fig). HDAC2 and 3 were found to be enriched at the *Ifng* promoter in the presence of HA-Foxp3 or HA-Foxp3^∆FKHnls^ while the enrichment was lost upon deletion of the ProR (S9C Fig) and therefore deacetylation did not occur for this mutant (S9D Fig).

Taken together, our data demonstrate that Foxp3 can be recruited to promoters in an FKH-independent fashion. The binding of Foxp3 leads to a ProR-dependent recruitment of class I HDACs, which in turn counteracts activation-induced hyperacetylation, thus inhibiting induction of gene expression.

## Discussion

Arguably the most important finding of our study is that the various domains and subdomains of Foxp3 fulfil distinct, discernible functions in gene regulation. The vast majority of Foxp3-regulated genes are affected by loss of the ProR. It has been previously suggested that structural flexibility caused by the high proline content of the ProR might be key to its role in complex formation with a multitude of interaction partners [43, 44]. Indeed, we find clusters of residues under purifying selection, which we believe to represent conserved binding sites for a variety of interaction partners while the proline residues at cluster boundaries provide flexibility for dynamic complex formation. Our finding that selective deletion of these putative subdomains affects different, largely non-overlapping sets of genes suggests that Foxp3 utilizes different mechanistic pathways to regulate gene expression. Our data imply that the ProR links Foxp3 to at least three independent mechanism of regulation: one involves the subdomain encoded by exon 1, another requires a four amino-acid motif within exon 2 (m4.2) and a third is a more complex association of class I HDACs with the entire ProR.

Surprisingly, the FKH appeared to be dispensable for the transcriptional control of most Foxp3 regulated T_Reg_ relevant genes. However, one has to be very cautious when extending our findings to draw conclusions regarding the relative importance to T_Reg_ cell function of individual functional domains of Foxp3. It would be naive to conclude that the FKH is less functionally important than any other domain, because its deletion ‘only’ dysregulates the expression of 16% (395) of Foxp3 regulated genes. Within this FKH-dependent set are genes with known T_Reg_ function such as *Ctla4*. A conditional knockout of this single gene within T_Reg_ cells results in a complete loss of regulatory function and an IPEX-like phenotype [45]. Furthermore, the greater proportion of immunology-related GO terms enriched within the lists of genes dysregulated by deletion of the FKH may reflect the fact that the role of the FKH is more focused on regulating a greater breadth of immune-related functions than the ProR. The first IPEX mutations described were located in the FKH domain of human Foxp3 [46] and we have demonstrated previously that T_H_::Foxp3^ΔFKH^ cells completely fail to compensate for the lack of T_Reg_ cells in mice [28]. Our findings here are consistent with IPEX mutations within the FKH domain causing pathological impairment of T_Reg_ function. IPEX mutations have now been found throughout Foxp3 with many of them occurring within the ProR [46, 47]. It is noteworthy that those IPEX mutations that destabilize Foxp3 on the mRNA or protein level have the most severe phenotypes as they lead to complete loss of Foxp3 function [46]. This is in agreement with our proposal that Foxp3 acts through multiple different mechanisms to exert its control upon distinct sets of genes.

We worried that deletion of the FKH may lead to an overestimation of the importance of this domain due to interference with nuclear localization of Foxp3. This was not the case. Despite lacking an NLS, Foxp3^ΔFKH^ was not completely excluded from the nucleus and retained the majority of its regulatory capacity. This is likely due to it being shuttled to the nucleus by other transcription factors as has been demonstrated for its interaction with c-Rel [21]. However, the regulation of some genes appears to be more sensitive to a lower concentration of Foxp3 in the nucleus than others. We also found that reintroduction of an SV40-derived NLS can have detrimental effects on Foxp3 function leading to a loss of the regulation of another set of genes. This suggests that the correct nuclear concentration of Foxp3 is important due to its participation in the dynamic formation of different complexes. Therefore, modifications altering the stability of Foxp3 such as its acetylation, phosphorylation and ubiquitylation play an important role in regulating gene expression and T_Reg_ function [48–50].

Contrary to published data [18], we demonstrate that the FKH, which interacts with both NFAT and DNA, is dispensable for the suppression of *Il2* expression. It cannot be ruled out that FKH point mutations or introduction of bulky residues described in this previous analysis [18] led to more widespread structural disturbances of Foxp3 and so affected more than just its ability to associate with NFAT through the FKH domain. Furthermore, the authors show that deletion of the Foxp3 N-terminal region (encompassing the ProR) dysregulates expression of IL-2, CD25 and CTLA-4 although a mechanism to explain this is not provided. We show that as long as sufficient Foxp3 is in the nucleus, a complex containing transcription factors and class I HDACs is formed. This does not preclude a role for FKH-mediated NFAT/Foxp3 interaction in *Il2* regulation, but demonstrates that it is not essential in the context of modulation of histone acetylation at the *Il2* promoter. Foxp3^ΔFKHnls^ is efficiently recruited to the *Il2* promoter. We propose that the FKH-independent interaction of Foxp3 with AML1 and/or c-Rel and/or AP-1 [19, 21, 40], possibly other factors mediates its recruitment to their binding sites in the IL2 promoter. Foxp3 can thus reverse the normally pro-transcriptional effects of DNA-bound transcription factors to ensure proper commitment to the T_Reg_ lineage.

It was previously shown that Foxp3 binding at the *Il2* and *Ifng* promoters coincides with their deacetylation [24]. Here we provide the full molecular mechanism for HDAC-mediated suppression of *Il2* and *Ifng* by Foxp3. We show that Foxp3 recruits class I HDACs to the promoters of *Il2* and *Ifng* in a ProR-dependent, FKH-independent manner. Furthermore, we demonstrate that this Foxp3-mediated suppression of *Il2* and *Ifng* in T_H_::Foxp3 cells is sensitive to inhibitors of class I HDACs or knockdown of class I HDACs. The fact that we were not able to define a specific interaction domain using co-immunoprecipitation of HDACs with Foxp3 mutants overexpressed in HEK293T cells implies a complex mode of interaction. Yet, we show using *in vitro* translated Foxp3 and bacterially-produced HDACs that class I HDACs interact directly with Foxp3. We propose that Foxp3 acts as a scaffold connecting DNA binding by its interaction partners to HDAC recruitment at promoters such as those of *Il2* and *Ifng*. This in turn leads to suppression of gene expression by counteracting activation-induced histone hyperacetylation. This mechanism should not be confused with the proposed function of class II HDAC in the regulation of Foxp3 expression and T_Reg_ cell function [23, 51]. Whilst class I HDACs are known to deacetylate histones, class IIa HDACs are enzymatically inactive and act primarily through recruiting other proteins [52]. Whilst it has been proposed that class II HDACs in combination with the histone acetylase TIP-60 are involved in IL-2 suppression [23], their role in this process remains unclear. It appears that TIP-60 acetylates Foxp3 itself [23], which suggests an altogether more indirect effect on IL-2 expression.

Unlike conventional CD4 helper T cells, T_Reg_ cells fail to remodel the *Il2* proximal promoter to become accessible chromatin upon stimulation [53]. This suggests that Foxp3-induced deacetylation of the *Il2* promoter is involved in establishing silent chromatin during T_Reg_ fate commitment. This establishment of epigenetic state is mimicked in T_H_::Foxp3 cells. We believe it is very likely that this mechanism of suppression is involved in the Foxp3-mediated suppression of many genes whose promoters would otherwise become hyperacetylated.

More broadly, Foxp3 may act as a bridge between any of its DNA-bound interaction partners (including AML1, c-Rel and AP-1) and other factors that modulate gene expression via chromatin modifications (eg HMTs, HATs, DNA demethylases). Foxp3 could thus affect the expression of a large number of genes. The fact that the promoters and enhancers of many Foxp3-regulated genes are not directly bound by Foxp3 [15, 17] suggests that, in many cases, this might be independent of Foxp3’s own DNA-binding abilities as we observe in the case of *Il2* and *Ifng*.

Since many of the genes that are functionally important in TReg cells are also transiently induced upon activation of T_H_ cells [15], we analysed the transcriptomes of cells rested after transduction with the Foxp3 constructs. Yet, it is clear that activation of T_Reg_ cells also changes their transcriptional profiles. Indeed, we observe this in the case of *Il2* and *Ifng* where Foxp3 counteracts activation-induced hyperacetylation. Further transcriptomic studies will be required to fully elucidate the additional biologically important layer of complexity that is introduced by signalling through the TCR *in vivo* [54].

We have devised a generalizable strategy to dissect Foxp3 on a molecular level and to analyse the function of individual domains and subdomains. We foresee that this approach will be useful in future studies by firstly suggesting a clear road map to further dissect Foxp3, secondly providing us with Foxp3 mutants only affecting a small number of candidate genes and thirdly allowing us to correlate Foxp3 subdomains with Foxp3 complex formation and function. This provides a pliable system in which to study lineage commitment of T_Reg_ cells as well as the functional roles of gene expression modules.

## Materials and Methods

### Mice and cell culture

BALB/c and B6.Foxp3(hCD2) mice[55] were maintained under specific pathogen-free conditions at the MRC-ARES animal facility (Cambridge, UK). CD4^+^CD25^-^ T cells were isolated from BALB/c mice between 2–4 months of age by negatively depleting splenocytes stained with FITC-conjugated depletion cocktail antibodies anti-CD8, anti-CD19, anti-CD11b, anti-CD11c, anti-Ly6G, anti-TER119 and anti-CD25 using an AutoMACS separator (Miltenyi Biotec). Isolated mouse primary T cells were cultured in RPMI-1640 medium supplemented with 10% FCS, 50 μg/mL gentamicin and 50 μM 2-mercaptoethanol. For *in vivo* experiments, no animals were excluded from the analyses and no statistical method was used to predetermine sample size. Age and sex-matched animals were randomly assigned to groups and technicians performing the work and assessing outcomes were blinded to the treatments. All experimental procedures received local ethics committee approval and were conducted in accordance with the Home Office Animals (Scientific Procedures) Act of 1986. Human embryonic kidney 293T cells (ATCC, USA) were used as the packaging cell line for producing viral particles and as the host for co-expressing proteins for co-immunoprecipitation experiments. These were cultured in IMDM supplemented with 10% FCS and 50 μg/mL gentamicin. All cells were cultured in a humidified incubator at 37^°^C, with 5% CO_2_.

### Antibodies

The antibodies used in this study are described in S4 Table.

### Expression vectors

For retroviral transduction of cells, cDNA encoding full-length Foxp3 (with HA or FLAG tag when necessary), Foxp3 deletion mutants, negative control GFP or HDACs 1, 2, 3 and 7 were cloned into the bicistronic MLV-based retroviral plasmid m6p co-expressing a GPI-linked extracellular part of ratCD8a (rCD8a) [56]. For *in vitro* translation, cDNA encoding Foxp3 was cloned into the pCDNA3.1(+) vector (Invitrogen). For expression in *E*. *coli*, cDNA encoding HDAC1, 2 and 3 was cloned into pGEX-6P-1 (Amersham).

### Virus production and T cell transduction

Virus supernatant was produced by transfecting 293T cells with equal amounts of retroviral vector and pCL-Eco packaging plasmid (Novus Biologicals) using the Phosphate Transfection Kit (Invitrogen). Supernatant of transfected cells was collected at 48 h and 72 h post-transfection and was snap-frozen in liquid nitrogen and stored at -20°C. For retroviral transduction, primary CD4+CD25- T cells were activated with plate-coated anti-CD3 (1 μg/ml) and soluble anti-CD28 (1 μg/ml) for 16 h. Reactivated cells were then resuspended in fresh cRPMI medium with an equal volume of viral supernatant and 5ng/ml murine IL-2 (Peprotech) and 8 μg/ml polybrene. After 12 h incubation at 37 C^o^, with 5% CO_2_, cells were harvested by centrifugation at 1260xg for 1.5 h at 32°C and transferred to fresh cRPMI supplemented with IL-2.

### RNA-seq library preparation and sequencing

Transduced CD4^+^CD25^-^ primary T cells were rested for 48h in RPMI medium supplemented with IL-2 (5 ng/ml). Rested cells expressing the transduction marker rCD8a were sorted by FACS to >95% purity. mRNA isolation and cDNA synthesis were performed using the uMACS mRNA isolation kit and uMACS cDNA synthesis module (Miltenyi Biotec). 5x10^5^ cells were used for each sample. Second strand synthesis was performed using *E*. *coli* DNA polymerase I (NEB), *E*. *coli* DNA ligase (NEB) and *E*. *coli* RNaseH (NEB) in second strand synthesis buffer (Invitrogen) at 16 °C for 2.5 h. Double-stranded DNA was fragmented by sonication using the Bioruptor UCD-400 (Diagenode), with 20 cycles of 30 s on, 30 s off. Fragmented samples were end-repaired using Klenow large fragment (NEB), T4 DNA polymerase (NEB) and T4 polynucleotide kinase (NEB) at 20°C for 30 min. DNA was purified using Zymo DNA Clean and Concentrator kits (Zymo). Purified blunt-ended DNA was 3’ adenylated using Klenow exo- fragment (NEB) at 37°C for 30 min. DNA was purified as before and adapters containing 6 bp barcodes from the sample prep oligonucleotide kit (Illumina) were ligated to 3’ A-tailed DNA fragment at 20°C for 30 min using T4 DNA ligase (NEB). Each DNA sample was amplified via 18-cycles of PCR using universal primers from the sample prep oligonucleotide kit, The PCR conditions are 30 sec at 98^°^C followed by 18 cycles of 10 sec at 98^°^C, 30 sec at 65^°^C, and 30 sec at 72^°^C, then a final step of 5mins at 72^°^C.The library was separated by agarose gel electrophoresis using a 2% gel and fragments between 200–400 bp were excised and purified using Minelute gel extraction kit (Qiagen). The concentration and size distribution of fragments was then determined with a Bioanalyzer High-Sensitivity DNA Chip (Agilent). RNA-seq libraries from different samples were multiplexed in equimolar concentrations for sequencing using a Hiseq 2000 (Illumina) at the Cancer Research Institute (Cambridge, UK).

### RNA-seq data analysis

Reads were aligned against GRCm38 mouse genome release 75 using the spliced-read mapper GSNAP [57]. GSNAP command line parameters were “-A sam-B 5-t 38-n 1-Q—-nofails” so that only uniquely-mapped reads were reported. The htseq-count Python script [58] was used to determine the number of reads from each sample that mapped to each feature within the GRCm38v75 GTF file. The script was run in “union” mode so that reads overlapping more than one feature were ignored. Transcript lengths for each gene were calculated from the sum of all annotated non-overlapping exonic regions. Robust FPKM estimates were calculated using the DESeq2 R package [59]. DESeq2 was also used to calculate regularized log-transformed expression counts. RNA-seq data for naive T_H_ and splenic T_Reg_ cells [29] were processed as for other data.

### Differential expression analysis

Genes were assigned to high (HE), low (LE), intermediate (INT) and non-expressing (NE) categories by performing expectation-maximization curve fitting of a Gaussian mixture model on log2-transformed FPKMs using the R package ‘MClust’ as described previously [27]. Kernel density estimates of read count distributions were performed with the density function available in R using the default settings apart from setting the bandwidth to be twice the default. HE and LE class boundaries were set for a false discovery rate of 0.05 as described for the EpiChIP software [60] although the classification procedure was, here, reimplemented in R. Genes that fell between the expression boundaries were classified as ‘intermediate’. DESeq2 was used to calculate moderated fold changes and associated adjusted *P* values (using Wald tests with Benjamini Hochberg correction for multiple testing) for all genes in every pairwise comparison between samples. A gene was defined as differentially expressed between two samples if: (i) It was classified as INT or HE in at least one sequencing replicate and (ii) it had an adjusted p value of less than 0.05. Genes were defined as Foxp3-regulated if they were differentially expressed in both Naive T_H_ cells vs splenic T_Reg_ and also in T_H_::control vs T_H_::Foxp3. A gene was defined as dysregulated in a particular condition if (i) it was initially defined as Foxp3-regulated as above, (ii) it was found to be differentially expressed between T_H_::Foxp3 and the condition of interest and (iii) its change in gene expression was in the direction of T_H_::control. Conversely, a gene was defined as maintaining its Foxp3-like regulation in a particular condition if (i) it was initially defined as Foxp3-regulated as above and (ii) it was not dysregulated.

### Clustering/PCA

Euclidean distances between samples were calculated from rlog transformed gene expression values for Foxp3-regulated genes (using the dist R function). Hierarchical clustering was performed using these distances with the heatmap.2 function (from the gplots R package). Principal component analysis was performed using rlog transformed gene expression values for Foxp3-regulated genes in each sample.

### Gene ontology enrichment and gene function analysis

The ‘Comprehensive list of Immune-Related Genes’ downloaded from https://immport.niaid.nih.gov [32, accessed 9^th^ April 2015] and mouse orthologs found using Ensemble BioMart (http://www.ensembl.org/biomart/). Genes that appeared in this list of orthologs were classified as ‘immunology-related’.

GO term enrichment analyses were performed using the tool available at http://amigo.geneontology.org/rte. Terms were considered enriched if they had a *P* value < 0.01. The manually-curated list of immunology keywords was downloaded from the ImmPort project and used to search the lists of enriched GO terms—terms containing a keyword were classified as ‘Immunology-related’.

### Antibody staining for flow cytometry and microscopic study

For cell surface antibody staining, cells were washed in PBS supplemented with 2% FBS and incubated with fluorophore conjugated antibodies (APC-αCD25, dilution V:V: 1:600; PE-αCD25, 1:600; PE-αrCD8a, 1:200; PB-αCD4, 1:400; Fc-Block (αCD16/32), 1:500; APC-αCD62L, 1:2500; APC-αLAG3, 1:200; FITC-αGITR, 1:300) at 4°C for 10 min. For intracellular staining, cells were then fixed and permeabilized using Foxp3 intracellular staining kit (eBioscience) before being stained with corresponding antibodies (APC-αCTLA4, 1:300; APC-αFoxp3 1:100; FITC-αHA, 1:250; FITC-αFLAG, 1:200; APC-αIL-2, 1:50; PE-αIgG2a, 1:100; FITC-αIgG2a, 1:250; APC-αIgG2a 1:100). After three washes in permeabilizing buffer stained cells were resuspended in PBS and analysed on an LSRII flow cytometer (BD Biosciences Immunocytometry Systems). Data were analyzed with FlowJo software. For localization analysis, Foxp3 and mutant transduced 293T cell were cultured on cover slides and fixed and permeabilized with Foxp3 Intracellular Staining Kit (eBioscience). After blocking with 5% BSA in PBS, cells were stained with AlexaFluor488-αFoxp3 antibody (Dilution 1:500 in permeabilization buffer for 2 h. After three washes using cell permeabilization buffer, cells were mounted on slides and imaged using a Zeiss LSM 710 confocal microscope.

### siRNA knockdown in primary T cells

Accell self-delivery siRNA (GE, Dharmacon siRNA) was used to knock down Class I HDACs. Foxp3 transduced T_H_ cells were treated with 1.5 μM control siRNA and a cocktail containing 1.5 μM HDAC1, 1.5 μM HDAC2 & 1.5 μM HDAC3 siRNA in siRNA delivery medium with 10 ng/ml IL2 and 2% FBS (GE, Dharmacon siRNA) for 96 h. Then the cells were re-stimulated with cell activation cocktail plus protein transport inhibitor for 12 h, permeabilized and stained for IL2.

### Comparative genomics

All sequences were retrieved from the NCBI, UCSC and Ensembl databases before October 2010. Protein sequences were aligned using Muscle as implemented in the Geneious program package [61]. The coiled coil, zinc finger and forkhead domains of Foxp3 were predicted using the SMART and COILS2 programs at http://smart.embl-heidelberg.de/smart/. The nuclear localization signal within the FKH was determined based on the presence or absence of a RKKR motif [33]. The REL test implemented on the Datamonkey website [62] was used with codon-aligned Foxp3 sequences from mouse, cat, cow, macaque, dog, horse, human, pig, rat, rhesus macaque and sheep. Prior to running the REL test, a model selection tool was executed to select the best nucleotide substitution model. All codons showing evidence of purifying selection with the REL test (based on non-synonymous over synonymous nucleotide replacements (dN>dS)) also replicated in an independent SLAC and FEL test. All three tests agreed when using a Bayes factor cutoff value of >40. The plots were generated using EMBOSS plotcon with a window size of 20.

### CFSE proliferation assay

CD4^+^CD25^-^ T_H_ cells were harvested by centrifugation for 10 min at 400xg and resuspended at 2x10^7^ cells/ml in prewarmed PBS in a 15mL falcon tube. The cells were then mixed 1:1 with pre-warmed 10 μM CFSE solution in PBS and incubated for 15 min at 37°C. Stained cells were washed twice with ice-cold PBS-FBS before being resuspended in pre-warmed cRPMI. Cells were then plated at 2x10^5^ cells per well in αCD3ε coated (0.6μg/ml) and soluble αCD28 (1μg/ml) U-bottomed 96-well plates together with 2x10^5^ Th cells transduced with wild-type Foxp3 or its mutants. Cells were cultured at 37°C, 5%CO2 and the proliferation of the labelled cells was assessed by determination CFSE dilution by FACS after 72 h.

### *In vivo* functional assessment of Foxp3 and mutants ectopic expression in T_H_ cells

A colitis induction model [63] was adopted for the assessment of the *in vivo* suppressive competence of T cells transduced with Foxp3 and its mutants. Three days prior to adoptive transfer, CD4^+^CD25^-^ were isolated from 8–10 week old wild-type Balb/c female mice. These cells were transduced with either wild type Foxp3, a mutant version of Foxp3 or the control. After transduction, cells were rested for one day in cRPMI supplemented with mIL-2 (5 ng/ml). On the day of adoptive transfer the transduced cells were FACS sorted to >98% purity and CD4^+^CD25^-^CD45RB^hi^ (>98% pure) T cells were isolated from spleens of 8–10 week old female Balb/c mice by MACS followed by FACS sorting. Weight-matched female C.B.-17 SCID mice between seven and eight weeks of age each received an intraperitoneal (i.p.) injection of 2x10^5^ CD4^+^CD25^-^CD45RB^hi^ cells along with 2x10^5^ transduced cells. The weight of the mice during the study was recorded by trained animal technicians at the MRC-ARES facility (Cambridge, UK). Mice reaching 20% weight loss (or defined welfare end-points) were humanely sacrificed. Five to seven weeks post-transfer the remaining mice were humanely sacrificed.

### Quantitative real-time PCR

Transduced cells were MACS sorted to >90% purity. Cells were either rested for 48 hours without activation or re-activated with plate-coated αCD3ε (5 μg/ml) and soluble αCD28 (1μg/ml) for 6 h. Total RNA was isolated using the RNeasy micro kit (Qiagen), and was reversely transcribed to cDNA with random hexamer priming using the SuperScript III kit (Invitrogen). The cDNA was used for quantitative PCR with SYBR green reagent (Thermo Scientific) according to manufacturer's instruction. Real-time qPCR was performed using the ABI/ViiA7 detection system (PE Applied Biosystems). Melting curves of the amplified products were obtained to determine the specificity of the amplification reaction. Cycling conditions were 2 min at 50°C, 5 min at 95°C followed by 40 cycles of 15s at 95°C, 60s at 60°C. Data was analyzed using the ΔΔCt method. For relative expression level detection, the data were normalized to CD3ε expression. For ChIP-qPCR, data were normalized to the input DNA. Primers were as detailed in S5 Table.

### Co-immunoprecipitation and western blotting

HEK293T cells were co-transfected with equal amounts of HA-tagged Foxp3 or its mutant plasmids and FLAG-tagged interaction protein plasmid using phosphate transfection kit (Invitrogen). 24 h post-transfection, 293T cells were lysed with hypertonic lysis buffer (20 mM Tris-HCl pH 7.4, 0.4 M NaCl, 1 mM EDTA, 1 mM EGTA, 1% Triton X-100, Roche Complete Protease Inhibitor Cocktail) and shaken for 15 min at 4°C. Cell lysate was pelleted by centrifugation for 15 min at 16000 g. The supernatant was collected and diluted with an equal volume of hypotonic dilution buffer (20 mM Tris-HCl pH 7.4, 1 mM EDTA, 1 mM EGTA, 0.1% Triton X-100, 20% glycerol, Roche Complete Protease Inhibitor Cocktail). 10% cell lysate was retained at -20°C for gene expression verification. The remaining lysate was incubated with αFLAG M2 Affinity Gel (Invitrogen) or αHA affinity gel (Invitrogen) for 2 h with rotation at 4°C. Unbound protein was removed by washing three times with high salt wash buffer (20 mM Tris-HCl pH7.4, 0.5 M NaCl, 1 mM EDTA, 1 mM EGTA, 0.1% Triton X-100, 10% glycerol, protease inhibitors). Precipitated protein was eluted from the affinity gel with 150 μg/ml 3XFLAG or 100 μg/ml HA peptide in PBS buffer. Protein samples were separated by SDS-PAGE using NuPAGE 4–12% bis-tris acrylamide gels. After electrophoresis, proteins were transferred from the gel to PVDF membrane (Millipore). After incubating with corresponding primary antibodies and HRP-conjugated secondary antibodies, the development of signal was performed using chemiluminescence ECL Standard Western Blotting Detection Reagents (GE). For co-IP experiments in primary T cells, CD4+CD25- TH cells were transduced with virus carrying the appropriate tag-fused protein followed by immunoprecipitation as described above.

### GST pull-down assay

BL21 chemically competent *E*. *coli* (NEB) were transformed with pGEX-6P-1 containing HDAC cDNA. The expression of GST-fused protein was induced by adding 0.1 mM IPTG followed by incubation for 6 h at 20°C. Cells were lysed using Bugbuster protein extraction reagent (Novagen) at RT for 20 min with gentle shaking. The lysate was centrifuged for 15 min at 16000xg at 4°C and the supernatant was mixed with glutathione sepharose 4B beads for overnight incubation at 4°C. The beads were washed five times with PBS and then eluted using 20 mM reduced glutathione. The protein sample was then desalted with a protein desalting column (Thermo Scientific). pCDNA3.1(+) plasmid containing Foxp3 cDNA was used as template for *in vitro* transcription and translation (TNT coupled reticulocyte lysate system, Promega). Five μl of *in vitro* synthesized protein was incubated with 10 μg of the affinity purified GST-tagged HDAC protein in BC100 buffer (20mM Tris pH7.9, 100mM NaCl, 10%glycerol, 0.2mM EDTA, 0.1% Triton X-100, complete protease inhibitor cocktail) on a rotator overnight at 4°C. The proteins were pulled down using Glutathione sepharose 4B beads, followed by five washes with BC100 buffer. The bound proteins were eluted with reduced SDS loading buffer and the eluted materials were resolved on SDS-PAGE. The presence of Foxp3 and GST-proteins were detected with αFoxp3 (Rat), αGST(Goat) and corresponding secondary antibodies.

### Chromatin-immunoprecipitation (ChIP)

Virus-transduced T_H_ cells were enriched using PE-αCD8a (1:200) together with αPE magnetic beads (Miltenyi Biotec) using the AutoMACS separator. Transduced cells were MACS sorted to >90% purity. The expression of HA tag and Foxp3 was confirmed by intracellular staining and flow cytometry. Cells were either rested 48 hours without activation or re-activated with plate-coated αCD3ε (5 μg/ml) and soluble αCD28 (1 μg/ml) for 6 hours. Chromatin- immunoprecipitation was performed using the EZ-ChIP kit (Millipore). Briefly, cells were fixed and protein/DNA was crosslinked in 1% formaldehyde (Sigma). After quenching with 0.125 M glycine, fixed cells were lysed in SDS lysis buffer and the lysate was sonicated to 200 to 600 bp fragments using the Bioruptor UCD-400 (Diagenode) with 20 cycles of 30 s On, 30 s off. After pre-clearing with protein G agarose beads (Invitrogen), sonicated samples were diluted in ChIP dilution buffer and incubated with either αIgG or corresponding ChIP-grade antibodies overnight. Protein G agarose beads were added to precipitate antibody-protein-DNA complexes. After five washes with the buffers provided by the manufacturer, the protein/DNA complex was eluted and the crosslinking was reversed by incubating at 65°C overnight. After digestion with RNase A and proteinase K to remove RNA and protein contents, the precipitated DNA was purified using QIAquick PCR purification kit (Qiagen). The purified DNA was either amplified by PCR reaction and analyzed in 2% agarose gel or quantified directly by qPCR. HA-GFP transduced cells served as control for non-specific binding in anti-HA ChIP and to determine the basal level in anti-HDAC and anti-Acetyl H3 ChIP. In anti-acetyl H3 ChIP of the resting and activated T cells, normal rabbit IgG was used as control for non-specific background signal.

### Accession codes

RNA-seq data: ArrayExpress E-MTAB-2988

## Supporting Information

S1 Fig Bimodal distributions of gene expression. (A) Curve fitting based on expectation maximisation was performed for each RNA-seq dataset (black line, kernel density estimate). Gaussian mixture models were fitted to identify the low expression (LE, orange line) and high expression (HE, purple line) peaks. The LE boundary (red dotted line) was calculated using an FDR of 0.05 with regards to the overlaps between LE and HE. (B) Bimodal distribution of gene expression in biological duplicates of each mutant.
(PDF)

S2 Fig Identification of T_Reg_-relevant, Foxp3-regulated genes. (A) Differentially expressed genes were identified for the comparison between naive T_H_ vs splenic T_Reg_ cells and also for T_H_::Foxp3 vs T_H_::control cells. The 2407 genes of interest for this work lie in the intersection between the two sets. (B) Prior microarray gene expression studies found fewer differentially expressed genes when comparing T_Reg_ and naive T_H_ cells (3092) [30]. These overlap well with our set of 2407 and using this set in our transcriptomic analyses does not alter our conclusions. (C) Schematic indicating the definition of genes that are dysregulated by ectopic expression of Foxp3 mutants. A gene was defined as dysregulated in a particular condition if (i) it was found in the intersection of sets in A above, (ii) it was found to be differentially expressed between T_H_::Foxp3 and the condition of interest and (iii) its change in gene expression was in the direction of T_H_::control. Conversely, a gene was defined as maintaining its Foxp3-like regulation in a particular condition if (i) it was initially defined as Foxp3-regulated as above and (ii) it was not dysregulated.
(PDF)

S3 Fig Expression of Foxp3 mutants. (A) Expression and localization of wild type Foxp3 and various subregion deletion mutant in Proline-rich domain. Upper: Intracellular staining of Foxp3 using anti-Foxp3 or anti-FLAG antibodies (the epitope recognized by the Foxp3 antibody is located in exon two and thus the expression of Foxp3 of deletion mutant ΔProR, Δe1-2, Δm4 and Δm4.2 can not be visualized by anti-Foxp3 staining. Instead, a FLAG-tag was added to the N-terminus of these mutants and their expression or localization was verified with a FLAG-specific antibody) in HEK293 cell transfected with the indicated constructs and analyzed by confocal microscope; Lower: FACS-plots of CD4^+^CD25^-^ T cells transduced with the indicated constructs double stained for the transduction marker rCD8a and Foxp3 or FLAG tag (Plots of ΔProR, Δe1-2, Δm4 and Δm4.2 were gated on rCD8a^+^ cells). (B) Alignment of mouse Foxp3 with Foxp3 orthologs from other placental and non-placental mammals as well as non-mammalian species. Proline-rich domain of placental orthologs were divided into 4 distinct regions (m1-m4) based on comparative genomics analysis, each of which is framed by proline residues.
(PDF)

S4 Fig Expression of Foxp3 protein levels in primary T_Reg_ cells and transduced T_H_ cells. FACS analysis of Foxp3 expression in (A) T_Reg_ cells from B6.Foxp3(hCD2) [56], (B) transduced CD25-depleted T_H_ cells (T_H_ cells) or (C) Foxp3-transduced CD25-depleted T_H_ cells (T_H_::Foxp3 cells). Total spleen lymphocytes were stained intracellularly with anti-Foxp3 or anti-hCD52 antibody and analyzed by FACS. Foxp3 expression levels within each gated population in A–C are shown as histograms in (D). Purple: CD4^+^CD25^-^ T_H_ cells; Blue: CD4^+^rCD8a^+^ T_H_::Foxp3 cells; Red: CD4^+^hCD2^+^ T_Reg_ cells.
(PDF)

S5 Fig Subcellular localization of Foxp3 mutants. HEK293 cells were transduced with either Foxp3, Foxp3^ΔFKH^ or Foxp3^ΔFKHnls^, stained with anti-Foxp3 antibody and DAPI and analyzed by confocal microscope.
(PDF)

S6 Fig Subdivisions of region m4. (A) Alignment of amino acids 60–82 of Foxp3 from mouse, rat, human, rhesus macaque, crab-eating macaque, cow, dog, cat, pig and horse. A graph indicating the average Bayes factor was overlaid and single amino acids with a Bayes factor higher than 40 are marked with red. A graph indicating the pairwise identity was overlaid in green. Prolines were marked with purple. The sequences of alanine replacement mutant ∆m4 as well as alanine replacement mutants ∆m4.1 and ∆m4.2, which narrow down the ∆m4 region, were shown below. (B) Quantitative real-time PCR analysis of the expression of T_Reg_ markers in CD4^+^CD25^-^ T cells transduced with the indicated constructs. The cells were kept on αCD3 activation for 36h during the virus transduction. Transduced cells expressing surface rCD8a were magnetically enriched and rested for 48 h before mRNA collection. In the case of IL-2, enriched cells were re-activation with CD3/CD28 for 6 h.
(PDF)

S7 Fig Additional data on Foxp3 class I HDAC interaction. (A) Foxp3 interacts with Class I HDACs in primary T cells. Primary CD4^+^ T cells were co-transduced with retrovirus carrying FLAG-Foxp3 and HA-HDAC1 or 2, or HA-Foxp3 and FLAG-HDAC3. Cell lysates were immunoprecipitated with anti-HA affinity gel (Left) and anti-FLAG M2 agarose (Right), followed by Western blotting with anti-HA or anti-FLAG antibodies, with anti-actin as loading control. (B) FLAG-HDAC1 (left), FLAG-HDAC2 (middle) or FLAG-HDAC3 (right) was co-transfected into 293T cells with the indicated HA-tagged WT Foxp3 and deletion constructs. Immunoprecipitation was performed with anti-FLAG M2 agarose. Precipitated proteins were probed with anti-HA (top), or anti-FLAG (middle) antibodies. The expression of Foxp3 and each mutant in cell lysate was detected by anti-HA antibody (bottom). (C) FACS-plots of CD4^+^CD25^-^ T cells transduced with HA-tagged Foxp3, double stained for the HA-tag and Foxp3. (D) Cells from (C) were rested for 48 h and re-activated by CD3/CD28 for 6 h. The resting and re-activated cells were subject to anti-HA or rabbit normal IgG ChIP. PCR (D) and qPCR (E) were performed to analyze the amount of chromatin precipitated using primers spanning proximal or distal parts of *Il2* promoters as illustrated in Fig 7A. P values in (E) were determined by one way analysis of variance (ANOVA) followed by Tukey’s post-hoc test (* indicates p<0.05; ** indicates p<0.01). (F-G) Foxp3 ectopic expression did not affect Class I HDAC expression. Primary CD4+CD25- T cells were transduced with HA-GFP control virus or Foxp3-IRES-rCD8a virus. rCD8a^+^ Cells were enriched by magnetic sorting (F) and the cell lysate was analyzed by immunoblotting using anti-Foxp3, anti-HDAC1, anti-HDAC2 and anti-HDAC3 antibody, with (G) anti-actin as loading control.
(PDF)

S8 Fig Class I HDAC siRNA dysregulates Foxp3 mediated IL-2 suppression. (A) Foxp3 transduced T_H_ cells were treated with either control siRNA or a cocktail containing HDAC1, HDAC2 and HDAC3 siRNA. (B) Foxp3 expression levels were determined in rCD8a^+^ (transduction reporter) cell population by intracellular Foxp3 staining. (C) Mean percentage (±SD, n = 4) of cells producing IL-2 in CD4^+^rCD8a^+^ cells under the various conditions shown in (A). *P* values were determined by ANOVA followed by Tukey’s post-hoc test (*** indicates *P*<0.001).
(PDF)

S9 Fig Foxp3 recruits class I HDAC to modulate histone acetylation of the *Ifng* promoter. (A) Expression of *Ifng* mRNA in Foxp3 or Foxp3 mutant transduced T_H_ cells at resting or re-activation state, determined by quantitative RT-PCR relative to the expression of *Cd3ε* (n = 2). (B) Primary T_H_ cells were transduced with HA-Foxp3, HA-GFP and HA-tagged Foxp3 deletion mutants. Transduced cells were rested and reactivated by CD3/CD28 for 6 h. ChIP-qPCR was used to analyze the binding of HA-GFP, HA-Foxp3 and deletion mutant at the *Ifng* promoter using anti-HA antibody (n = 2). (C) ChIP-qPCR analysis of the binding of Class I HDACs at the *Ifng* promoter in re-activated transduced cells expressing HA-tagged GFP, Foxp3 or the respective deletion mutant (n = 3). (D) ChIP-qPCR analysis of *Ifng* promoter in re-activated transduced cells expressing HA-tagged GFP, Foxp3 or the respective deletion mutant using anti-acetylated histone 3 antibody (n = 3). P values in (B-D) between HA-GFP and HA-Foxp3 or the respective Foxp3 mutant transduced cells were determined by one-way ANOVA followed by Tukey’s post-hoc test.
(PDF)

S1 Table Number of paired sequence reads. (DOC)

S2 Table Gene dysregulation in Foxp3 mutant transduced T_H_ cells. (XLSX)

S3 Table Gene ontology terms enriched within gene lists. (XLSX)

S4 Table Antibodies, cytokines and chemicals. (DOCX)

S5 Table PCR primers. (DOCX)

## References

[1] Wing K, Sakaguchi S (2010) Regulatory T cells exert checks and balances on self tolerance and autoimmunity. Nat Immunol 11: 7–13. 10.1038/ni.1818 20016504

[2] Kim JM, Rasmussen JP, Rudensky AY (2007) Regulatory T cells prevent catastrophic autoimmunity throughout the lifespan of mice. Nat Immunol 8: 191–197. 17136045 10.1038/ni1428

[3] Lahl K, Loddenkemper C, Drouin C, Freyer J, Arnason J, et al (2007) Selective depletion of Foxp3(+) regulatory T cells induces a scurfy-like disease. J. Exp. Med. 204: 57–63. 17200412 10.1084/jem.20061852 PMC2118432

[4] Chen T, Darrasse-Jeze G, Bergot AS, Courau T, Churlaud G, et al (2013) Self-specific memory regulatory T cells protect embryos at implantation in mice. J Immunol 191: 2273–2281. 10.4049/jimmunol.1202413 23913969 PMC4107421

[5] Izcue A, Coombes JL, Powrie F (2006) Regulatory T cells suppress systemic and mucosal immune activation to control intestinal inflammation. Immunol Rev 212: 256–271. 16903919 10.1111/j.0105-2896.2006.00423.x

[6] Campbell DJ, Ziegler SF (2007) FOXP3 modifies the phenotypic and functional properties of regulatory T cells. Nat Rev Immunol 7: 305–310. 17380159 10.1038/nri2061

[7] Fontenot JD, Rasmussen JP, Williams LM, Dooley JL, Farr AG, et al (2005) Regulatory T cell lineage specification by the forkhead transcription factor foxp3. Immunity 22: 329–341. 15780990 10.1016/j.immuni.2005.01.016

[8] Dhamne C, Chung Y, Alousi AM, Cooper LJ, Tran DQ (2013) Peripheral and thymic foxp3(+) regulatory T cells in search of origin, distinction, and function. Front Immunol 4: 253 10.3389/fimmu.2013.00253 23986762 PMC3753660

[9] Ohkura N, Hamaguchi M, Morikawa H, Sugimura K, Tanaka A, et al (2012) T Cell Receptor Stimulation-Induced Epigenetic Changes and Foxp3 Expression Are Independent and Complementary Events Required for Treg Cell Development. Immunity 37: 785–799. 10.1016/j.immuni.2012.09.010 23123060

[10] Brunkow ME, Jeffery EW, Hjerrild KA, Paeper B, Clark LB, et al (2001) Disruption of a new forkhead/winged-helix protein, scurfin, results in the fatal lymphoproliferative disorder of the scurfy mouse. Nat Genet 27: 68–73. 11138001 10.1038/83784

[11] Lahl K, Mayer CT, Bopp T, Huehn J, Loddenkemper C, et al (2009) Nonfunctional Regulatory T Cells and Defective Control of Th2 Cytokine Production in Natural Scurfy Mutant Mice. J. Immunl. 183: 5662–5672. 10.4049/jimmunol.0803762 19812199

[12] Li B, Samanta A, Song X, Iacono KT, Brennan P, et al (2007) FOXP3 is a homo-oligomer and a component of a supramolecular regulatory complex disabled in the human XLAAD/IPEX autoimmune disease. Int Immunol 19: 825–835. 17586580 10.1093/intimm/dxm043

[13] Rudra D, deRoos P, Chaudhry A, Niec RE, Arvey A, et al (2012) Transcription factor Foxp3 and its protein partners form a complex regulatory network. Nat Immunol 13: 1010–1019. 10.1038/ni.2402 22922362 PMC3448012

[14] Bandukwala HS, Wu Y, Feuerer M, Chen Y, Barboza B, et al (2011) Structure of a domain-swapped FOXP3 dimer on DNA and its function in regulatory T cells. Immunity 34: 479–491. 10.1016/j.immuni.2011.02.017 21458306 PMC3085397

[15] Marson A, Kretschmer K, Frampton GM, Jacobsen ES, Polansky JK, et al (2007) Foxp3 occupancy and regulation of key target genes during T-cell stimulation. Nature 445: 931–935. 17237765 10.1038/nature05478 PMC3008159

[16] Song X, Li B, Xiao Y, Chen C, Wang Q, et al (2012) Structural and biological features of FOXP3 dimerization relevant to regulatory T cell function. Cell Rep 1: 665–675. 10.1016/j.celrep.2012.04.012 22813742 PMC3401381

[17] Zheng Y, Josefowicz SZ, Kas A, Chu TT, Gavin MA, et al (2007) Genome-wide analysis of Foxp3 target genes in developing and mature regulatory T cells. Nature 445: 936–940. 17237761 10.1038/nature05563

[18] Wu Y, Borde M, Heissmeyer V, Feuerer M, Lapan AD, et al (2006) FOXP3 controls regulatory T cell function through cooperation with NFAT. Cell 126: 375–387. 16873067 10.1016/j.cell.2006.05.042

[19] Ono M, Yaguchi H, Ohkura N, Kitabayashi I, Nagamura Y, et al (2007) Foxp3 controls regulatory T-cell function by interacting with AML1/Runx1. Nature 446: 685–689. 17377532 10.1038/nature05673

[20] Pan F, Yu H, Dang EV, Barbi J, Pan X, et al (2009) Eos mediates Foxp3-dependent gene silencing in CD4+ regulatory T cells. Science 325: 1142–1146. 10.1126/science.1176077 19696312 PMC2859703

[21] Loizou L, Andersen KG, Betz AG (2011) Foxp3 interacts with c-Rel to mediate NF-kappaB repression. PLoS One 6: e18670 10.1371/journal.pone.0018670 21490927 PMC3072406

[22] Zhang F, Meng G, Strober W (2008) Interactions among the transcription factors Runx1, RORgammat and Foxp3 regulate the differentiation of interleukin 17-producing T cells. Nat Immunol 9: 1297–1306. 10.1038/ni.1663 18849990 PMC4778724

[23] Li B, Samanta A, Song X, Iacono KT, Bembas K, et al (2007) FOXP3 interactions with histone acetyltransferase and class II histone deacetylases are required for repression. Proc Natl Acad Sci U S A 104: 4571–4576. 17360565 10.1073/pnas.0700298104 PMC1838642

[24] Chen C, Rowell EA, Thomas RM, Hancock WW, Wells AD (2006) Transcriptional regulation by Foxp3 is associated with direct promoter occupancy and modulation of histone acetylation. J Biol Chem 281: 36828–36834. 17028180 10.1074/jbc.M608848200

[25] Bettelli E, Dastrange M, Oukka M (2005) Foxp3 interacts with nuclear factor of activated T cells and NF-kappa B to repress cytokine gene expression and effector functions of T helper cells. Proc Natl Acad Sci U S A 102: 5138–5143. 15790681 10.1073/pnas.0501675102 PMC555574

[26] Andersen KG, Butcher T, Betz AG (2008) Specific immunosuppression with inducible Foxp3-transduced polyclonal T cells. PLoS Biol 6: e276 10.1371/journal.pbio.0060276 18998771 PMC2581628

[27] Hebenstreit D, Fang M, Gu M, Charoensawan V, van Oudenaarden A, et al (2011) RNA sequencing reveals two major classes of gene expression levels in metazoan cells. Mol Syst Biol 7: 497 10.1038/msb.2011.28 21654674 PMC3159973

[28] Andersen KG, Nissen JK, Betz AG (2012) Comparative Genomics Reveals Key Gain-of-Function Events in Foxp3 during Regulatory T Cell Evolution. Front Immunol 3: 113 10.3389/fimmu.2012.00113 22590469 PMC3349156

[29] Stubbington MJT, Mahata B, Svensson V, Deonarine A, Nissen JK, et al (2015) An atlas of mouse CD4+ T cell transcriptomes. Biology Direct. 10:14 10.1186/s13062-015-0045-x 25886751 PMC4384382

[30] Samstein RM, Arvey A, Josefowicz SZ, Peng X, Reynolds A, et al (2012) Foxp3 Exploits a Pre-Existent Enhancer Landscape for Regulatory T Cell Lineage Specification. Cell 151: 153–166. 10.1016/j.cell.2012.06.053 23021222 PMC3493256

[31] Wang Z, Gerstein M, Snyder M (2009) RNA-Seq: a revolutionary tool for transcriptomics. Nat Rev Genet 10: 57–63. 10.1038/nrg2484 19015660 PMC2949280

[32] Bhattacharya S, Andorf S, Gomes L, Dunn P, Schaefer H, et al (2014) ImmPort: disseminating data to the public for the future of immunology. Immunol. Res. 58: 234–239. 10.1007/s12026-014-8516-1 24791905

[33] Hancock WW, Ozkaynak E (2009) Three Distinct Domains Contribute to Nuclear Transport of Murine Foxp3. Plos One 4: e7890 10.1371/journal.pone.0007890 19924293 PMC2774276

[34] Lin W, Haribhai D, Relland LM, Truong N, Carlson MR, et al (2007) Regulatory T cell development in the absence of functional Foxp3. Nat. Immunol. 8: 359–368. 17273171 10.1038/ni1445

[35] Camperio C, Caristi S, Fanelli G, Soligo M, Del Porto P, et al (2012) Forkhead Transcription Factor FOXP3 Upregulates CD25 Expression through Cooperation with RelA/NF-kappa B. Plos One 7: e48303 10.1371/journal.pone.0048303 23144749 PMC3483148

[36] Metzler B, Burkhart C, Wraith DC (1999) Phenotypic analysis of CTLA-4 and CD28 expression during transient peptide-induced T cell activation in vivo. Int Immunol 11: 667–675. 10330272 10.1093/intimm/11.5.667

[37] de la Rosa M, Rutz S, Dorninger H, Scheffold A (2004) Interleukin-2 is essential for CD4+CD25+ regulatory T cell function. Eur J Immunol 34: 2480–2488. 15307180 10.1002/eji.200425274

[38] Boucheron N, Tschismarov R, Goeschl L, Moser MA, Lagger S, et al (2014) CD4(+) T cell lineage integrity is controlled by the histone deacetylases HDAC1 and HDAC2. Nat Immunol 15: 439–448. 10.1038/ni.2864 24681565 PMC4346201

[39] Baine I, Basu S, Ames R, Sellers RS, Macian F (2013) Helios induces epigenetic silencing of IL2 gene expression in regulatory T cells. J Immunol 190: 1008–1016. 10.4049/jimmunol.1200792 23275607 PMC3558938

[40] Lee SM, Gao B, Fang DY (2008) FoxP3 maintains Treg unresponsiveness by selectively inhibiting the promoter DNA-binding activity of AP-1. Blood 111: 3599–3606. 10.1182/blood-2007-09-115014 18223166

[41] Bandyopadhyay S, Dure M, Paroder M, Soto-Nieves N, Puga I, et al (2007) Interleukin 2 gene transcription is regulated by Ikaros-induced changes in histone acetylation in anergic T cells. Blood 109: 2878–2886. 17148585 10.1182/blood-2006-07-037754 PMC1852212

[42] Bandyopadhyay S, Montagna C, Macian F (2012) Silencing of the Il2 gene transcription is regulated by epigenetic changes in anergic T cells. Eur J Immunol 42: 2471–2483. 10.1002/eji.201142307 22684523 PMC3681531

[43] Kay BK, Williamson MP, Sudol P (2000) The importance of being proline: the interaction of proline-rich motifs in signaling proteins with their cognate domains. Faseb Journal 14: 231–241. 10657980

[44] van der Lee R, Buljan M, Lang B, Weatheritt RJ, Daughdrill GW, et al (2014) Classification of Intrinsically Disordered Regions and Proteins. Chem Rev 114: 6589–6631. 10.1021/cr400525m 24773235 PMC4095912

[45] Sojka DK, Hughson A, Fowell DJ (2009) CTLA-4 is required by CD4(+)CD25(+) Treg to control CD4(+) T-cell lymphopenia-induced proliferation. Eur J Immunol 39: 1544–1551. 10.1002/eji.200838603 19462377 PMC2835300

[46] Bachetta R, Gambineri E, Passerini L, Barzaghi F, Mannurita SC, et al (2012) Ipex Syndrome: Clinical and Immunological Findings. Update from the Italian Study Group of Ipex. J Clin Immunol 32: 79–79.

[47] Katoh H, Zheng P, Liu Y (2013) FOXP3: Genetic and epigenetic implications for autoimmunity. J Autoimmun 41: 72–78. 10.1016/j.jaut.2012.12.004 23313429 PMC3622774

[48] van Loosdregt J, Vercoulen Y, Guichelaar T, Gent YYJ, Beekman JM, et al (2010) Regulation of Treg functionality by acetylation-mediated Foxp3 protein stabilization. Blood 115: 965–974. 10.1182/blood-2009-02-207118 19996091

[49] Chen ZJ, Barbi J, Bu SR, Yang HY, Li ZY, et al (2013) The Ubiquitin Ligase Stub1 Negatively Modulates Regulatory T Cell Suppressive Activity by Promoting Degradation of the Transcription Factor Foxp3. Immunity 39: 272–285. 10.1016/j.immuni.2013.08.006 23973223 PMC3817295

[50] Morawski PA, Mehra P, Chen CX, Bhatti T, Wells AD (2013) Foxp3 Protein Stability Is Regulated by Cyclin-dependent Kinase 2. J Biol Chem 288: 24494–24502. 10.1074/jbc.M113.467704 23853094 PMC3750148

[51] Tao R, de Zoeten EF, Ozkaynak E, Chen CX, Wang LQ, et al (2007) Deacetylase inhibition promotes the generation and function of regulatory T cells. Nat Med 13: 1299–1307. 17922010 10.1038/nm1652

[52] Akimova T, Beier UH, Liu YJ, Wang LQ, Hancock WW (2012) Histone/protein deacetylases and T-cell immune responses. Blood 119: 2443–2451. 10.1182/blood-2011-10-292003 22246031 PMC3311271

[53] Su L, Creusot RJ, Gallo EM, Chan SM, Utz PJ, et al (2004) Murine CD4(+) CD25(+) regulatory T cells fail to undergo chromatin remodeling across the proximal promoter region of the IL-2 gene. J Immunol 173: 4994–5001. 15470042 10.4049/jimmunol.173.8.4994

[54] Levine AG, Arvey A, Jin W, Rudensky AY (2014) Continuous requirement for the TCR in regulatory T cell function. Nat Immunol 15: 1070–1078. 10.1038/ni.3004 25263123 PMC4205268

[55] Kendal AR, Chen Y, Regateiro FS, Ma J, Adams E, et al (2011) Sustained suppression by Foxp3+ regulatory T cells is vital for infectious transplantation tolerance. J Exp Med. 208: 2043–2053. 10.1084/jem.20110767 21875958 PMC3182049

[56] Bloor S, Ryzhakov G, Wagner S, Jonathan P, Butler G, et al (2008) Signal processing by its coil zipper domain activates IKK gamma. Proc Natl Acad Sci U S A 105: 1279–1284. 10.1073/pnas.0706552105 18216269 PMC2234129

[57] Wu TD, Nacu S (2010) Fast and SNP-tolerant detection of complex variants and splicing in short reads. Bioinformatics 26: 873–881. 10.1093/bioinformatics/btq057 20147302 PMC2844994

[58] Anders S, Pyl PT, Huber W (2014) HTSeq—A Python framework to work with high-throughput sequencing data. *bioRxiv*. 10.1093/bioinformatics/btu638 PMC4287950 25260700

[59] Love MI, Huber W, Anders S (2014) Moderated estimation of fold change and dispersion for RNA-Seq data with DESeq2. *bioRxiv*. 10.1186/s13059-014-0550-8 PMC4302049 25516281

[60] Hebenstreit D, et al (2011) EpiChIP: gene-by-gene quantification of epigenetic modification levels. Nucleic Acids Res 39: e27 10.1093/nar/gkq1226 21131282 PMC3061070

[61] Kearse M, Moir R, Wilson A, Stones-Havas S, Cheung M, et al (2012) Geneious Basic: An integrated and extendable desktop software platform for the organization and analysis of sequence data. Bioinformatics 28: 1647–1649. 10.1093/bioinformatics/bts199 22543367 PMC3371832

[62] Pond SLK, Frost SDW (2005) Datamonkey: rapid detection of selective pressure on individual sites of codon alignments. Bioinformatics 21: 2531–2533. 15713735 10.1093/bioinformatics/bti320

[63] Liu H, Hu B, Xu D, Liew FY (2003) CD4+CD25+ regulatory T cells cure murine colitis: the role of IL-10, TGF-beta, and CTLA4. J Immunol 171: 5012–5017. 14607897 10.4049/jimmunol.171.10.5012

