# Supplementary figures and images for: The Regulatory T Cell Lineage Factor Foxp3 Regulates Gene Expression through Several Distinct Mechanisms Mostly Independent of Direct DNA Binding
Source: PLoS Genet. 2015 Jun 24;11(6):e1005251. doi: 10.1371/journal.pgen.1005251 (PMC4480970; doi:10.1371/journal.pgen.1005251)

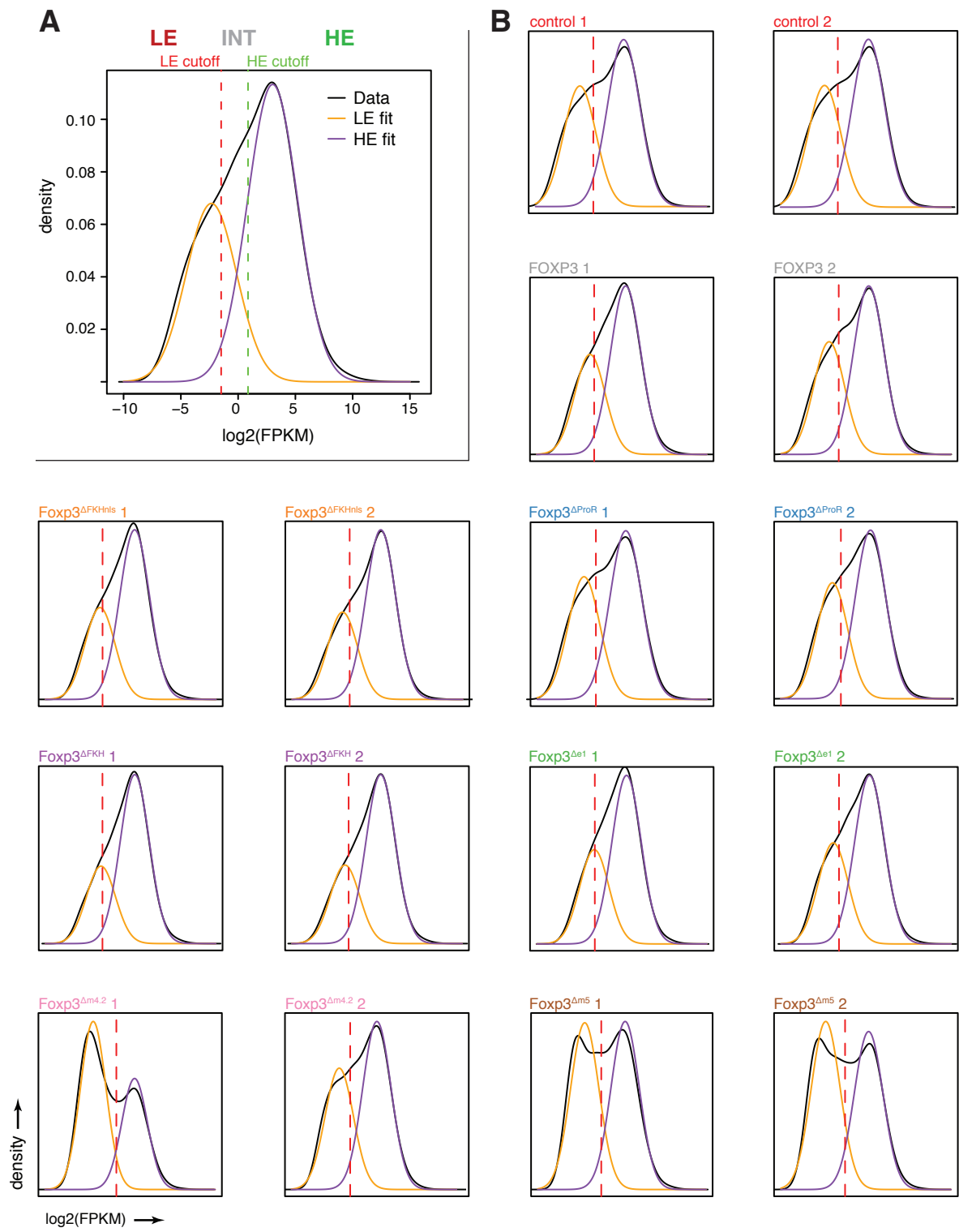

Supplement: S1 Fig — (A) Curve fitting based on expectation maximisation was performed for each RNA-seq dataset (black line, kernel density estimate). Gaussian mixture models were fitted to identify the low expression (LE, orange line) and high expression (HE, purple line) peaks. The LE boundary (red dotted line) was calculated using an FDR of 0.05 with regards to the overlaps between LE and HE. (B) Bimodal distribution of gene expression in biological duplicates of each mutant. (PDF) [file pgen.1005251.s001.pdf]

**A**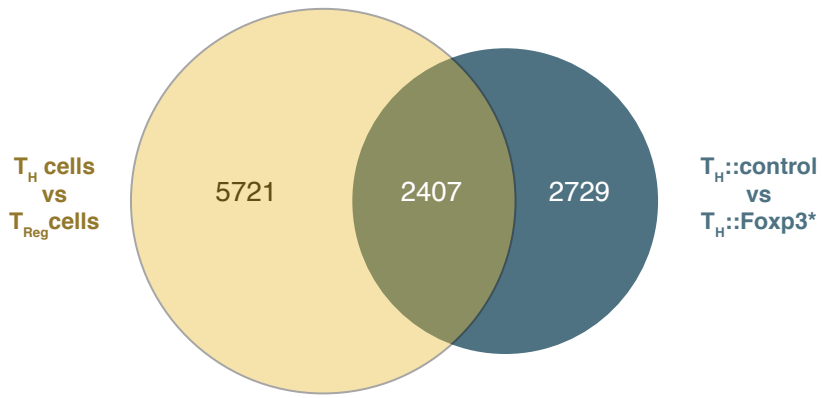**B**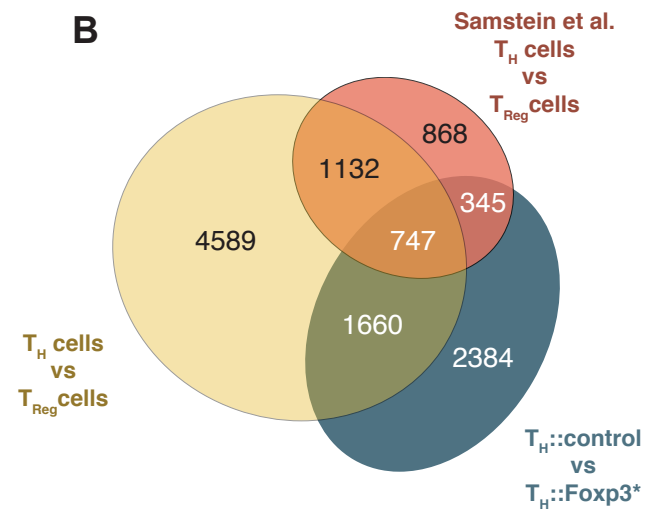**C**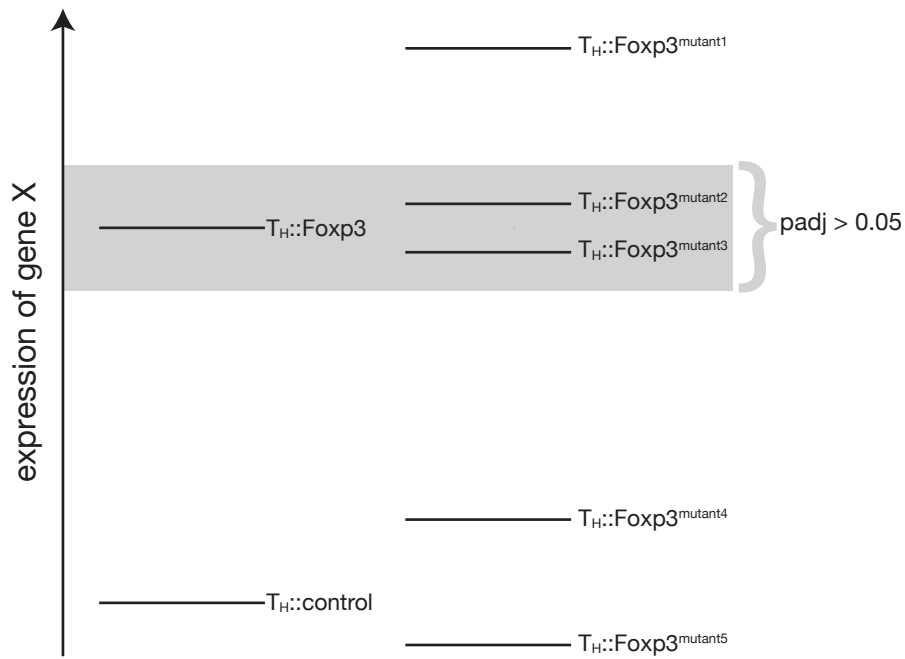

|         | differentially expressed | dysregulated |
|---------|--------------------------|--------------|
| mutant1 | ✓                        | ✗            |
| mutant2 | ✗                        | ✗            |
| mutant3 | ✗                        | ✗            |
| mutant4 | ✓                        | ✓            |
| mutant5 | ✓                        | ✓            |

Supplement: S2 Fig — (A) Differentially expressed genes were identified for the comparison between naive TH vs splenic TReg cells and also for TH::Foxp3 vs TH::control cells. The 2407 genes of interest for this work lie in the intersection between the two sets. (B) Prior microarray gene expression studies found fewer differentially expressed genes when comparing TReg and naive TH cells (3092) [30]. These overlap well with our set of 2407 and using this set in our transcriptomic analyses does not alter our conclusions. (C) Schematic indicating the definition of genes that are dysregulated by ectopic expression of Foxp3 mutants. A gene was defined as dysregulated in a particular condition if (i) it was found in the intersection of sets in A above, (ii) it was found to be differentially expressed between TH::Foxp3 and the condition of interest and (iii) its change in gene expression was in the direction of TH::control. Conversely, a gene was defined as maintaining its Foxp3-like regulation in a particular condition if (i) it was initially defined as Foxp3-regulated as above and (ii) it was not dysregulated. (PDF) [file pgen.1005251.s002.pdf]

**A**

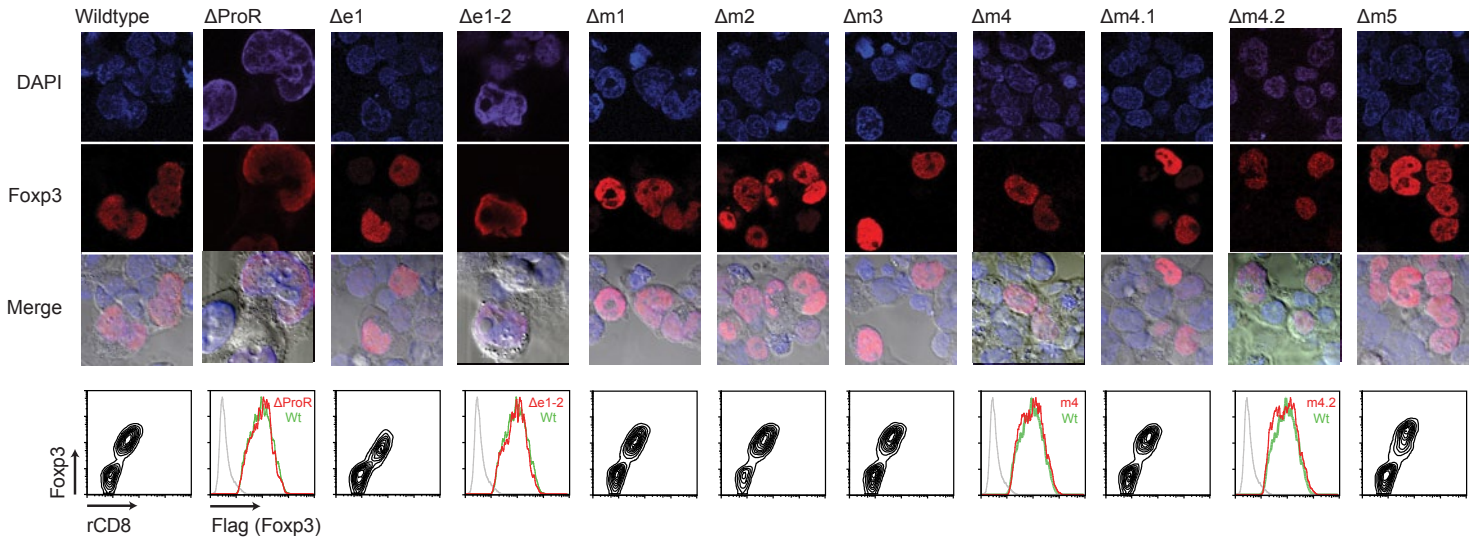

**B**

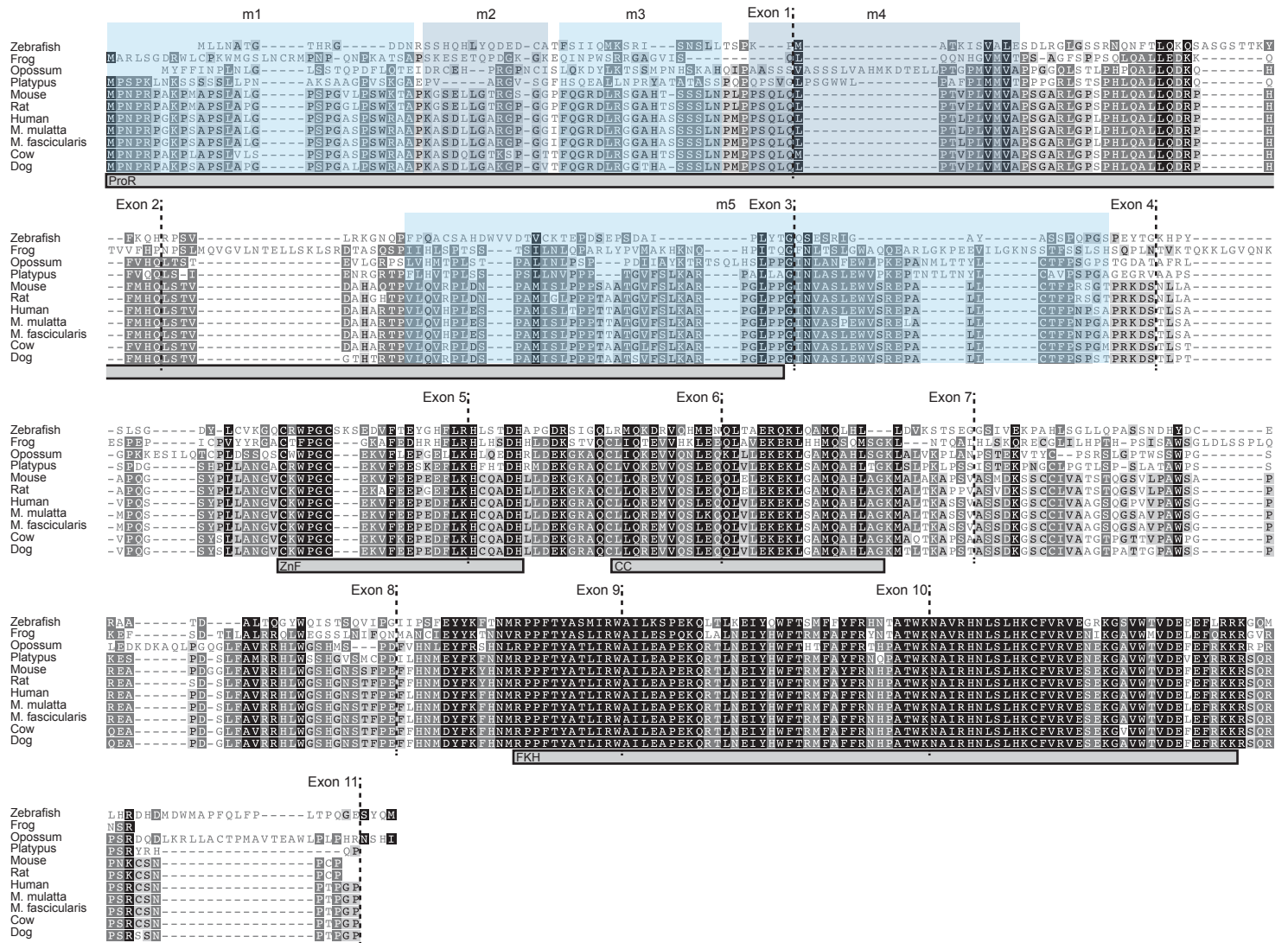

Supplement: S3 Fig — (A) Expression and localization of wild type Foxp3 and various subregion deletion mutant in Proline-rich domain. Upper: Intracellular staining of Foxp3 using anti-Foxp3 or anti-FLAG antibodies (the epitope recognized by the Foxp3 antibody is located in exon two and thus the expression of Foxp3 of deletion mutant ΔProR, Δe1-2, Δm4 and Δm4.2 can not be visualized by anti-Foxp3 staining. Instead, a FLAG-tag was added to the N-terminus of these mutants and their expression or localization was verified with a FLAG-specific antibody) in HEK293 cell transfected with the indicated constructs and analyzed by confocal microscope; Lower: FACS-plots of CD4+CD25- T cells transduced with the indicated constructs double stained for the transduction marker rCD8a and Foxp3 or FLAG tag (Plots of ΔProR, Δe1-2, Δm4 and Δm4.2 were gated on rCD8a+ cells). (B) Alignment of mouse Foxp3 with Foxp3 orthologs from other placental and non-placental mammals as well as non-mammalian species. Proline-rich domain of placental orthologs were divided into 4 distinct regions (m1-m4) based on comparative genomics analysis, each of which is framed by proline residues. (PDF) [file pgen.1005251.s003.pdf]

T<sub>H</sub> cells

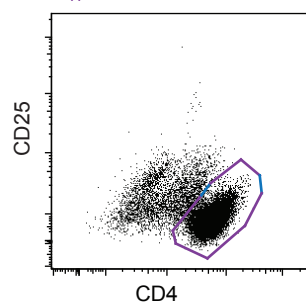

T<sub>H</sub>::Foxp3 cells

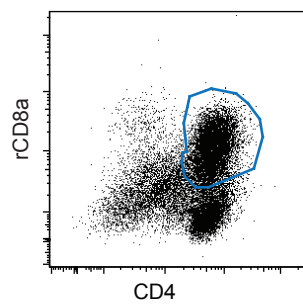

T<sub>reg</sub> cells

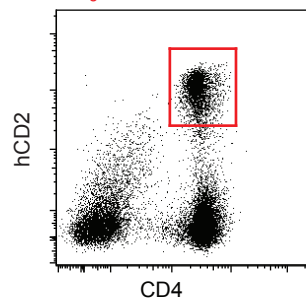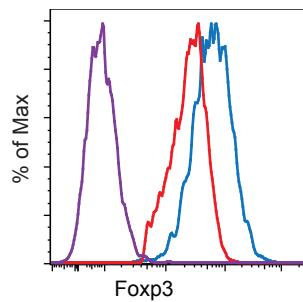

Supplement: S4 Fig — FACS analysis of Foxp3 expression in (A) TReg cells from B6.Foxp3(hCD2) [56], (B) transduced CD25-depleted TH cells (TH cells) or (C) Foxp3-transduced CD25-depleted TH cells (TH::Foxp3 cells). Total spleen lymphocytes were stained intracellularly with anti-Foxp3 or anti-hCD52 antibody and analyzed by FACS. Foxp3 expression levels within each gated population in A–C are shown as histograms in (D). Purple: CD4+CD25- TH cells; Blue: CD4+rCD8a+ TH::Foxp3 cells; Red: CD4+hCD2+ TReg cells. (PDF) [file pgen.1005251.s004.pdf]

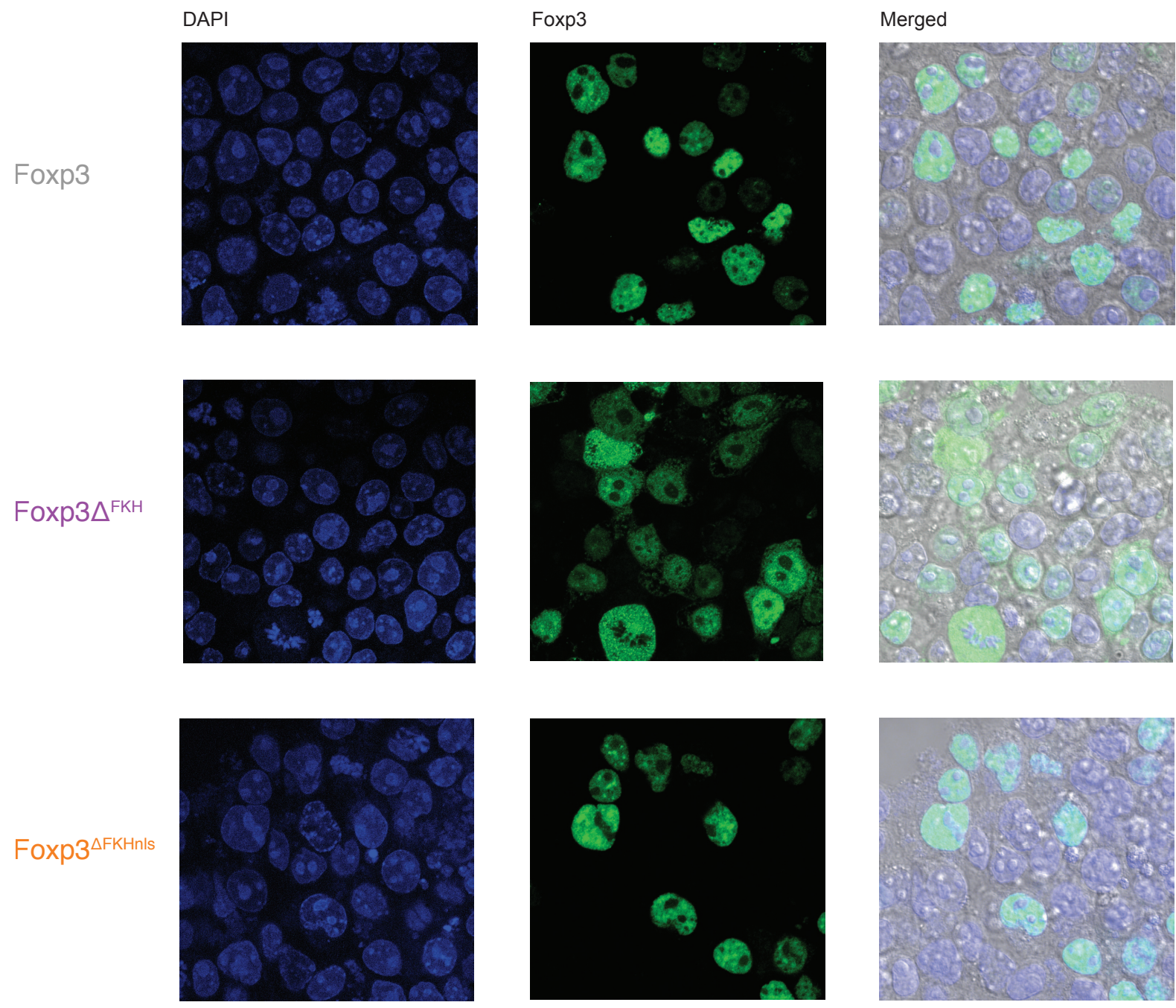

Supplement: S5 Fig — HEK293 cells were transduced with either Foxp3, Foxp3ΔFKH or Foxp3ΔFKHnls, stained with anti-Foxp3 antibody and DAPI and analyzed by confocal microscope. (PDF) [file pgen.1005251.s005.pdf]

A

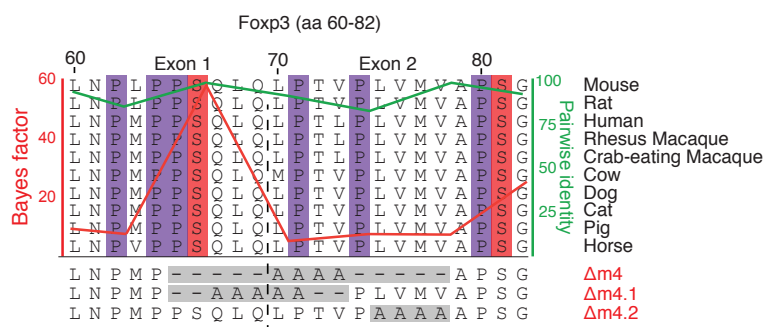

B

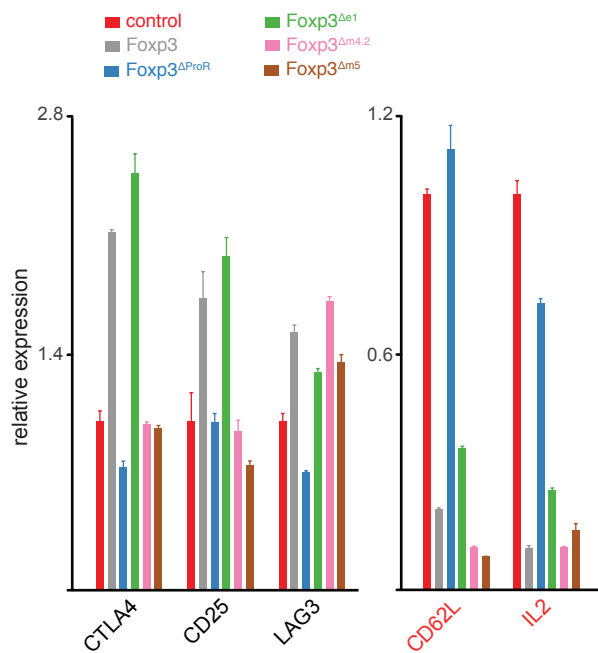

Supplement: S6 Fig — (A) Alignment of amino acids 60–82 of Foxp3 from mouse, rat, human, rhesus macaque, crab-eating macaque, cow, dog, cat, pig and horse. A graph indicating the average Bayes factor was overlaid and single amino acids with a Bayes factor higher than 40 are marked with red. A graph indicating the pairwise identity was overlaid in green. Prolines were marked with purple. The sequences of alanine replacement mutant ∆m4 as well as alanine replacement mutants ∆m4.1 and ∆m4.2, which narrow down the ∆m4 region, were shown below. (B) Quantitative real-time PCR analysis of the expression of TReg markers in CD4+CD25- T cells transduced with the indicated constructs. The cells were kept on αCD3 activation for 36h during the virus transduction. Transduced cells expressing surface rCD8a were magnetically enriched and rested for 48 h before mRNA collection. In the case of IL-2, enriched cells were re-activation with CD3/CD28 for 6 h. (PDF) [file pgen.1005251.s006.pdf]

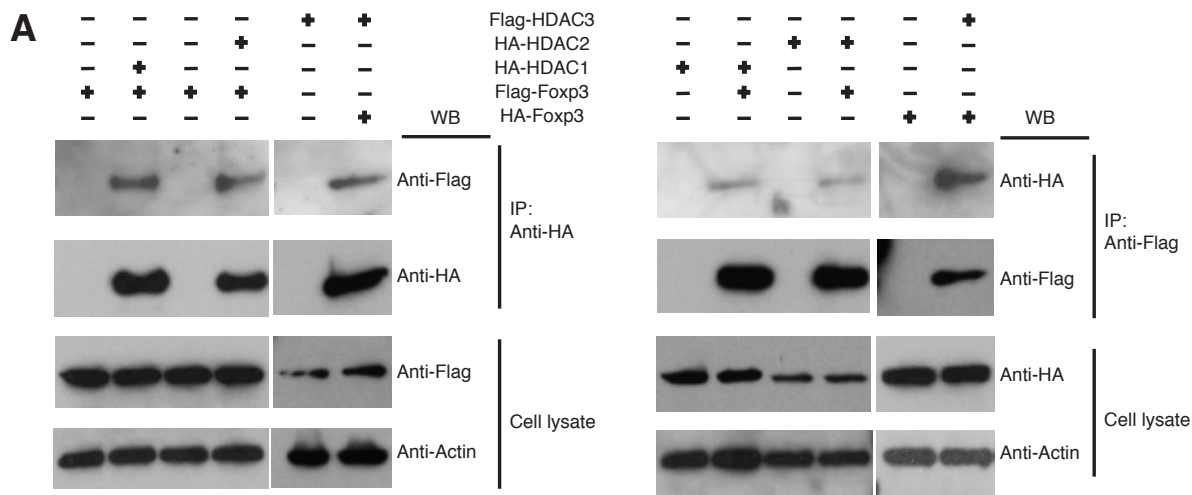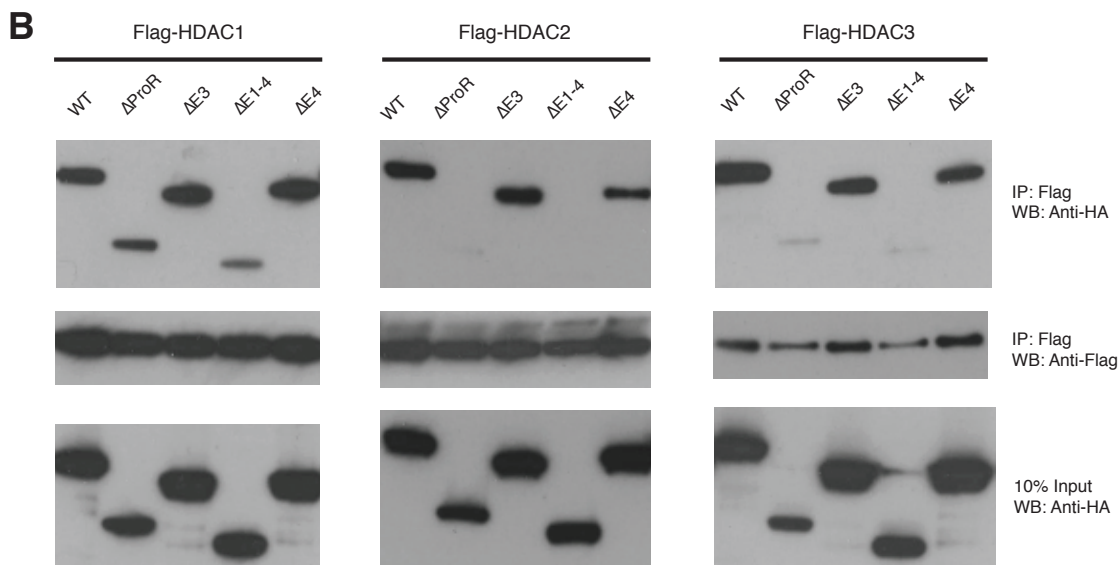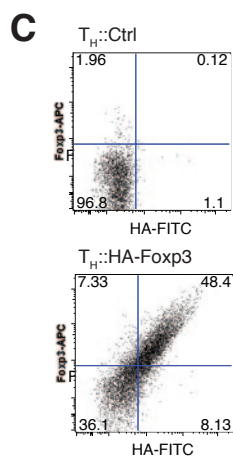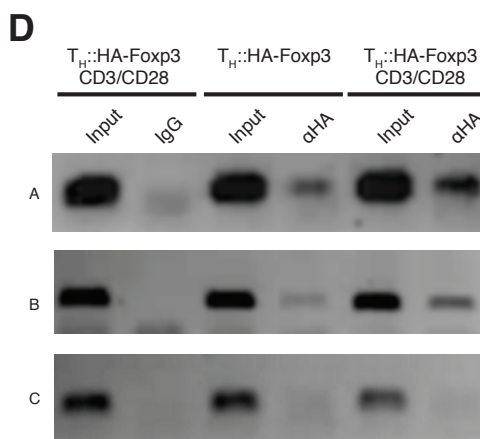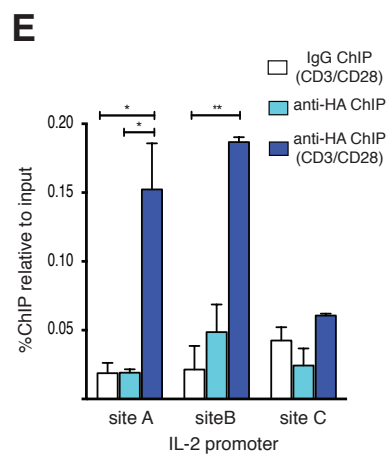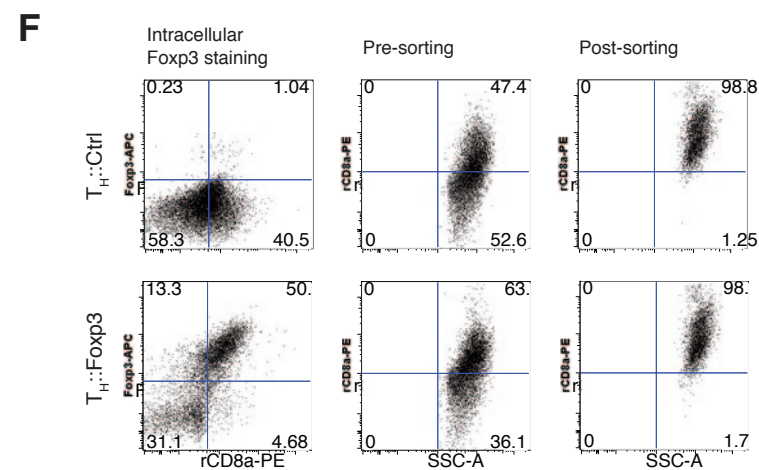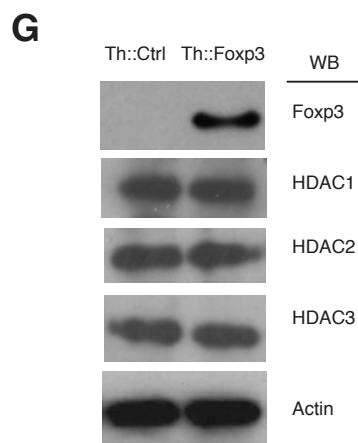

Supplement: S7 Fig — (A) Foxp3 interacts with Class I HDACs in primary T cells. Primary CD4+ T cells were co-transduced with retrovirus carrying FLAG-Foxp3 and HA-HDAC1 or 2, or HA-Foxp3 and FLAG-HDAC3. Cell lysates were immunoprecipitated with anti-HA affinity gel (Left) and anti-FLAG M2 agarose (Right), followed by Western blotting with anti-HA or anti-FLAG antibodies, with anti-actin as loading control. (B) FLAG-HDAC1 (left), FLAG-HDAC2 (middle) or FLAG-HDAC3 (right) was co-transfected into 293T cells with the indicated HA-tagged WT Foxp3 and deletion constructs. Immunoprecipitation was performed with anti-FLAG M2 agarose. Precipitated proteins were probed with anti-HA (top), or anti-FLAG (middle) antibodies. The expression of Foxp3 and each mutant in cell lysate was detected by anti-HA antibody (bottom). (C) FACS-plots of CD4+CD25- T cells transduced with HA-tagged Foxp3, double stained for the HA-tag and Foxp3. (D) Cells from (C) were rested for 48 h and re-activated by CD3/CD28 for 6 h. The resting and re-activated cells were subject to anti-HA or rabbit normal IgG ChIP. PCR (D) and qPCR (E) were performed to analyze the amount of chromatin precipitated using primers spanning proximal or distal parts of Il2 promoters as illustrated in Fig 7A. P values in (E) were determined by one way analysis of variance (ANOVA) followed by Tukey’s post-hoc test (* indicates p<0.05; ** indicates p<0.01). (F-G) Foxp3 ectopic expression did not affect Class I HDAC expression. Primary CD4+CD25- T cells were transduced with HA-GFP control virus or Foxp3-IRES-rCD8a virus. rCD8a+ Cells were enriched by magnetic sorting (F) and the cell lysate was analyzed by immunoblotting using anti-Foxp3, anti-HDAC1, anti-HDAC2 and anti-HDAC3 antibody, with (G) anti-actin as loading control. (PDF) [file pgen.1005251.s007.pdf]

**A**

resting

activated

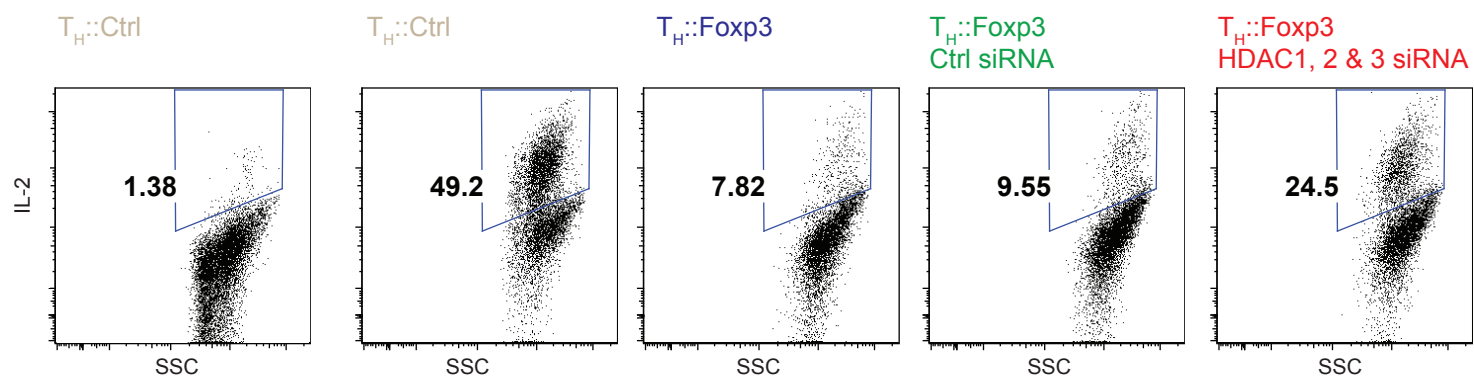**B**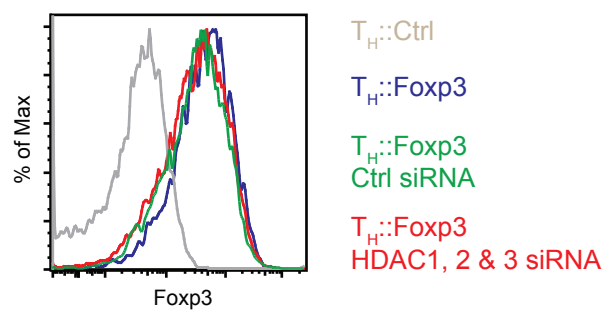**C**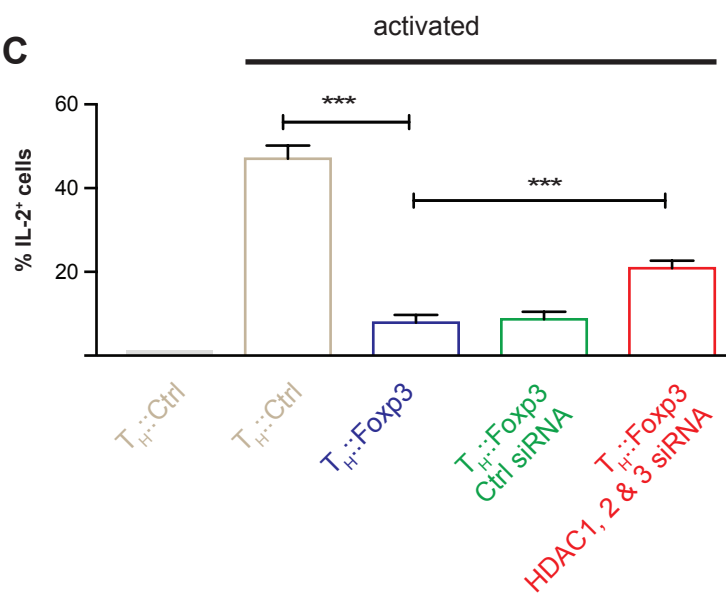

Supplement: S8 Fig — (A) Foxp3 transduced TH cells were treated with either control siRNA or a cocktail containing HDAC1, HDAC2 and HDAC3 siRNA. (B) Foxp3 expression levels were determined in rCD8a+ (transduction reporter) cell population by intracellular Foxp3 staining. (C) Mean percentage (±SD, n = 4) of cells producing IL-2 in CD4+rCD8a+ cells under the various conditions shown in (A). P values were determined by ANOVA followed by Tukey’s post-hoc test (*** indicates P<0.001). (PDF) [file pgen.1005251.s008.pdf]

**A**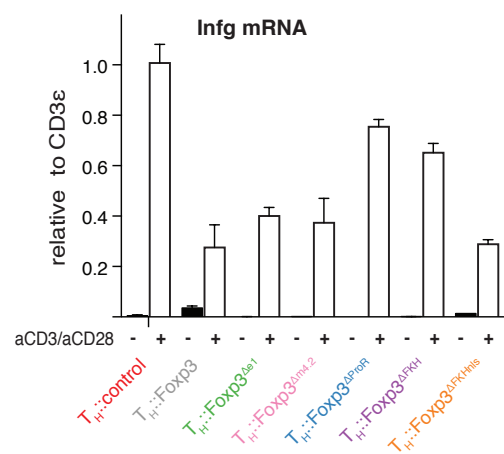**B**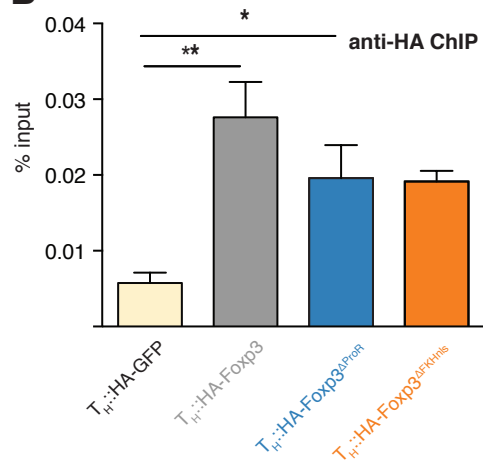**C**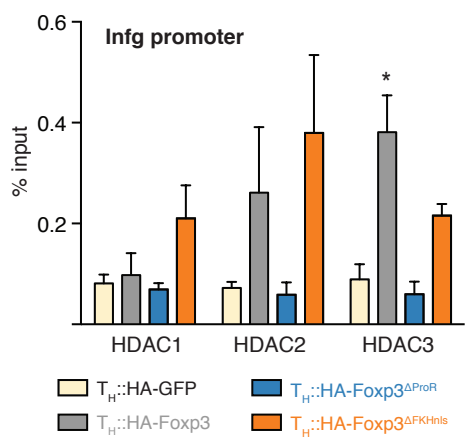**D**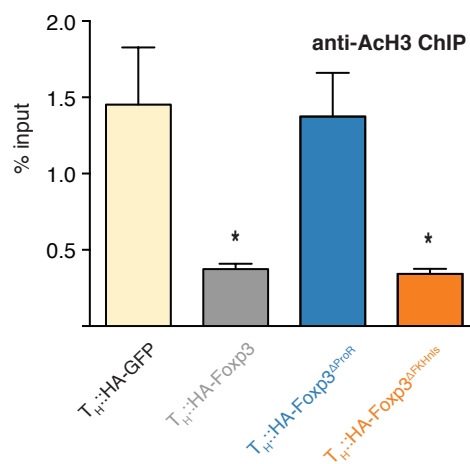

Supplement: S9 Fig — (A) Expression of Ifng mRNA in Foxp3 or Foxp3 mutant transduced TH cells at resting or re-activation state, determined by quantitative RT-PCR relative to the expression of Cd3ε (n = 2). (B) Primary TH cells were transduced with HA-Foxp3, HA-GFP and HA-tagged Foxp3 deletion mutants. Transduced cells were rested and reactivated by CD3/CD28 for 6 h. ChIP-qPCR was used to analyze the binding of HA-GFP, HA-Foxp3 and deletion mutant at the Ifng promoter using anti-HA antibody (n = 2). (C) ChIP-qPCR analysis of the binding of Class I HDACs at the Ifng promoter in re-activated transduced cells expressing HA-tagged GFP, Foxp3 or the respective deletion mutant (n = 3). (D) ChIP-qPCR analysis of Ifng promoter in re-activated transduced cells expressing HA-tagged GFP, Foxp3 or the respective deletion mutant using anti-acetylated histone 3 antibody (n = 3). P values in (B-D) between HA-GFP and HA-Foxp3 or the respective Foxp3 mutant transduced cells were determined by one-way ANOVA followed by Tukey’s post-hoc test. (PDF) [file pgen.1005251.s009.pdf]
