# Supplementary material for: The Regulatory T Cell Lineage Factor Foxp3 Regulates Gene Expression through Several Distinct Mechanisms Mostly Independent of Direct DNA Binding
Source: PLoS Genet. 2015 Jun 24;11(6):e1005251. doi: 10.1371/journal.pgen.1005251 (PMC4480970; doi:10.1371/journal.pgen.1005251)
Supplement: S1 Table — (DOC) [file pgen.1005251.s010.doc]

| **Sample** | **Total reads** | **Properly paired** | **% Properly paired** |
| --- | --- | --- | --- |
| control A | 119093381 | 102236235 | 85.8 |
| control B | 134981956 | 117063731 | 86.7 |
| dE1_A | 20052091 | 17062852 | 85.1 |
| dE1_B | 40601155 | 33492270 | 82.5 |
| dProR_A | 85266347 | 73495605 | 86.2 |
| dProR_B | 43256260 | 37338411 | 86.3 |
| dFKH_A | 28572803 | 23851971 | 83.5 |
| dFKH_B | 40833794 | 34182339 | 83.7 |
| dFKHnls_A | 31190649 | 25380617 | 81.4 |
| dFKHnls_B | 52344130 | 43801724 | 83.7 |
| FOXP3_A | 36239611 | 30881573 | 85.2 |
| FOXP3_B | 75490941 | 62515223 | 82.8 |
| m4p2_A | 74589604 | 44917216 | 60.2 |
| m4p2_B | 168497038 | 134421570 | 79.8 |
| m5_A | 140227984 | 65806742 | 46.9 |
| m5_B | 130093580 | 100731476 | 77.4 |
|  |  |  |  |
