# Supplementary material for: The Regulatory T Cell Lineage Factor Foxp3 Regulates Gene Expression through Several Distinct Mechanisms Mostly Independent of Direct DNA Binding
Source: PLoS Genet. 2015 Jun 24;11(6):e1005251. doi: 10.1371/journal.pgen.1005251 (PMC4480970; doi:10.1371/journal.pgen.1005251)
Supplement: S4 Table — (DOCX) [file pgen.1005251.s013.docx]

| **Source** | **Antibodies, cytokines and chemicals - Cat#** |
| --- | --- |
| Abcam | αHA (Rabbit ChIP grade-ab9110); αHDAC1 (Rabbit ChIP grade-ab7028); αHDAC2 (Rabbit ChIP grade-ab7029); αHDAC3 (Rabbit ChIP grade-ab7030); αAcH3 (Rabbit ChIP grade-ab47915) |
| BD Bioscience | FITC-αLy6G (Rat anti-mouse-551460); PE-αrCD8a (mouse anti-rat-554857); FITC-αCD4 (Rat anti-mouse-561835); FITC-αCD8a (Rat anti-mouse-553030); FITC-αCD25 (Rat anti-mouse-558689); FITC-αCD11b (Rat anti-mouse-561688); FITC-αCD11c (Hamster anti-mouse-553801); FITC-αCD19 (Rat anti-mouse-553785) |
| Cell signaling | αHDAC1 (Rabbit anti-mouse-5356); αHDAC2 (Rabbit anti-mouse-5113); αHDAC3 (Rabbit anti-mouse-2632) |
| Dako | HRP-αIgG (Rabbit anti-goat-P044901-2); HRP-αIgG (Goat anti-mouse-P-044701-2); HRP-αIgG (Rabbit anti-rat-P045001-2); HRP-αIgG (Goat anti-rabbit-P044901-2) |
| eBioscience | αCD28 (Rat anti-mouse-16-02-8186); αCD3ε (Hamster anti mouse-16-0031-85); APC-αCD25 (Rat anti-mouse-17-0251-82); APC-αCD62L (Rat anti-mouse-17-0621-82); APC-αLAG3 (Rat anti-mouse-17-2231-82); FITC-GITR (Rat anti-mouse-11-5874-82), V450-αCD4 (Rat anti-mouse-48-0042-82); APC-αCTLA-4 (Rat anti-mouse-17-1522-82); APC-αIL-2 (Rat anti-mouse-17-7021-82); APC-αFoxp3 (Rat anti-mouse-17-5773-82); PE-αIgG1 (Rat anti-mouse-12-4015-82); PE-αIgG2a (Rat anti-mouse-12-4321-81); FITC-αIgG2a (Rat anti-mouse-11-4321-80); APC-αIgG2a (Rat anti-mouse-17-4732-42); PE-αIgG2b (Rat anti-mouse-12-4724-81) |
| Fisher Scientific | αGST (Goat-PA1-32120) |
| LKT Laboratories | Entinostat (Class I HDACi-E5477) |
| Millipore | normal mouse IgG (12-371); normal rabbit IgG (12-370); Histone Deacetylase Inhibitor VII 106 (Class I HDACi-382173) |
| Peprotech | Murine IL2 (212-12) |
| Santa Cruz | αACTIN (Rabbit anti-mouse rat & Human-sc1616) |
| Sigma | FITC-αFLAG (Rabbit-F4049); FITC-αHA (Rabbit-H7411); HRP-αFLAG (Mouse-A8592); Trichostatin A (TSA-T8552); Isopropyl b-D-1-thiogalactopyranoside (IPTG-I6758); L-Glutathione Reduced (G4251); Lysozyme (L6876); Phenylmethanesulfonylfluoride (PMSF-P7626); Polybrene (H9268); Protein G Sepharose Fast Flow (P3296); 3X Flag Tag (F4799); Influenza Hemagglutinin (I2149); Anti-Flag M2 affinity gel (A2220); EZview Red Anti-HA Affinity Gel (E6779) |
| Roche | HRP- αHA (Mouse-12013819001) |
| Torcris Bioscience | MC1568 (Class II HDACi-4077) |
