# Supplementary material for: The Regulatory T Cell Lineage Factor Foxp3 Regulates Gene Expression through Several Distinct Mechanisms Mostly Independent of Direct DNA Binding
Source: PLoS Genet. 2015 Jun 24;11(6):e1005251. doi: 10.1371/journal.pgen.1005251 (PMC4480970; doi:10.1371/journal.pgen.1005251)
Supplement: S5 Table — (DOCX) [file pgen.1005251.s014.docx]

| Primers for qPCR | | |
| --- | --- | --- |
| CD25 | forward: 5’ AACCATAGTACCCAGTTGTCGG3’  reverse:5’ TCCTAAGCAACGCATATAGACCA3’ | |
| CTLA-4 | forward: 5’ TGTTGACACGGGACTGTACCT3’  reverse:5’ CGGGCATGGTTCTGGATCA3’ | |
| CD62L | forward: 5’ TACATTGCCCAAAAGCCCTTAT3’  reverse:5’ CATCGTTCCATTTCCCAGAGTC3’ | |
| LAG3 | forward: 5’ CTGGGACTGCTTTGGGAAG3’  reverse:5’ GGTTGATGTTGCCAGATAACCC3’ | |
| IL2 | forward: 5’ TGAGCAGGATGGAGAATTACAGG 3’  reverse: 5’ GTCCAAGTTCATCTTCTAGGCAC 3’ | |
| CD3ε | forward: 5’ ATGCGGTGGAACACTTTCTGG 3’  reverse: 5’ GCACGTCAACTCTACACTGGT 3’ | |
| IFNgamma | forward: 5’ ATGAACGCTACACACTGCATC 3’  reverse: 5’ CCATCCTTTTGCCAGTTCCTC 3’ | |
| Primers for ChIP-PCR or qPCR | | |
| IL2 promoter A | | forward: 5’ TCATACAGAAGGCGTTCATTGTATG 3’  reverse: 5’ CCTCTTCTGATGACTCTCTGGAAT 3’ |
| IL2 promoter B | | forward: 5’ CTTAGGGAGCTTGCCTGTGGT 3’  reverse: 5’ GTAGGTGTGTGGTGAGGCTTATG 3’ |
| IL2 promoter C | | forward: 5’ CATGCAGAGTTTTTTGTTGTTTTCTAG 3’  reverse: 5’ GCCTAAAGTCTCTCACAAAGAACAGA 3’ |
| CD3ε promoter | | forward: 5’ TTCCTGCCTCCGCTGGAGGG 3’  reverse: 5’ GGCAGAAGCCTCCGCCTTGG 3’ |
| IFNgamma promoter | | forward: 5’ CAGAGAATCCCACAAGAATGGCAC 3’  reverse: 5’ GGGGGAGCTAAGTTACTTTGCATTAC 3’ |
